# Mice Deficient in *Sfrp1* Exhibit Increased Adiposity, Dysregulated Glucose Metabolism, and Enhanced Macrophage Infiltration

**DOI:** 10.1371/journal.pone.0078320

**Published:** 2013-12-05

**Authors:** Kelly J. Gauger, Lotfi M. Bassa, Elizabeth M. Henchey, Josephine Wyman, Brooke Bentley, Melissa Brown, Akihiko Shimono, Sallie S. Schneider

**Affiliations:** 1 Pioneer Valley Life Sciences Institute, Baystate Medical Center, Springfield, Massachusetts, United States of America; 2 Biology Department, University of Massachusetts, Amherst, Massachusetts, United States of America; 3 Veterinary and Animal Sciences, University of Massachusetts, Amherst, Massachusetts, United States of America; 4 Department of Nutrition, University of Massachusetts, Amherst, Massachusetts, United States of America; 5 TransGenicInc., Kobe, Japan; University of Minnesota - Twin Cities, United States of America

## Abstract

The molecular mechanisms involved in the development of obesity and related complications remain unclear. Wnt signaling plays an important role in preadipocyte differentiation and adipogenesis. The expression of a Wnt antagonist, secreted frizzled related protein 1 (SFRP1), is increased in response to initial weight gain, then levels are reduced under conditions of extreme obesity in both humans and animals. Here we report that loss of *Sfrp1* exacerbates weight gain, glucose homeostasis and inflammation in mice in response to diet induced obesity (DIO). Sfrp1^-/-^ mice fed a high fat diet (HFD) exhibited an increase in body mass accompanied by increases in body fat percentage, visceral white adipose tissue (WAT) mass, and adipocyte size. Moreover, *Sfrp1* deficiency increases the mRNA levels of key *de novo* lipid synthesis genes (*Fasn*, *Acaca*, *Acly*, *Elovl*, *Scd1*) and the transcription factors that regulate their expression (*Lxr-α, Srebp1, Chreb*, and *Nr1h3*) in WAT. Fasting glucose levels are elevated, glucose clearance is impaired, hepatic gluconeogenesis regulators are aberrantly upregulated (*G6pc* and *Pck1*), and glucose transporters are repressed (*Slc2a2* and *Slc2a4*) in Sfrp1^-/-^ mice fed a HFD. Additionally, we observed increased steatosis in the livers of Sfrp1^-/-^ mice. When there is an expansion of adipose tissue there is a sustained inflammatory response accompanied by adipokine dysregulation, which leads to chronic subclinical inflammation. Thus, we assessed the inflammatory state of different tissues and revealed that Sfrp1^-/-^ mice fed a HFD exhibited increased macrophage infiltration and expression of pro-inflammatory markers including *IL-6, Nmnat, Tgf-β2*, and *SerpinE1*. Our findings demonstrate that the expression of *Sfrp1* is a critical factor required for maintaining appropriate cellular signaling in response to the onset of obesity.

## Introduction

Obesity is a severe epidemic that causes predisposition to several metabolic diseases such as cardiovascular diseases and type-2 diabetes. It is caused by an increase in adipocyte size and eventually an increase in preadipocyte differentiation due to excess diet intake [1,2]. In 2010, the United States of America CDC reported that 35.7% of adults and 17% of children in america are obese as [3]. This epidemic resulted in the push for more research aimed at exploring adipocyte differentiation and metabolism in order to further the understanding of molecular mechanisms which regulate adipocyte biology in order to develop potential therapeutic targets. 

Dietary fatty acids are primarily stored in white adipose tissue (WAT) as triglycerides and excess carbohydrates are converted to fats via *de novo* lipogenesis [4]. Though most cells perform *de novo* lipogenesis, adipocytes and hepatocytes are especially well adapted to the process. The genes involved in *de novo* lipogenesis are controlled by two master transcription regulator proteins which are regulated by either glucose (Carbohydrate Response Element Binding Protein -ChREBP) or insulin (Sterol Response Element Binding Protein 1- SREBP1). Increased *de novo* lipogenesis during adipogenesis is associated with improved insulin sensitivity [5], but the converse occurs in the liver where *de novo* lipogenesis correlates with insulin resistance, an increase in serum triglycerides, and adipocyte accumulation causing steatosis and nonalcoholic fatty liver disease [6].

The WAT produces adipokines, such as Interleukin-6 (IL-6), adiponectin, and tumor necrosis factor (TNFα) [7] as well as hormones including leptin, estrogen, and resistin. All of these factors play a variety of roles through different signaling pathways, including the Wnt/β-catenin signaling pathway, in multiple tissues ranging from promoting or inhibiting adipogenesis, regulating the immunoresponse and insulin sensitization. Activation of Wnt signaling occurs when Wnt ligands bind to Frizzled (FZD) receptors in conjunction with one of the LDL receptor-related proteins (LRP5 or LRP6). Receptor activation leads to increased nuclear β-catenin levels where it complexes with the TCF/LEF1 family of HMG box transcription factors and stimulates the expression of specific target genes. Wnt/β-catenin signaling has been shown to regulate adipogenesis in both paracrine and autocrine manners and to control adipocyte differentiation [8–10]. While Wnt6, Wnt10a and Wnt10b have been shown to inhibit preadipocyte differentiation [8–10], Wnt5b and Wnt4 promote adipogenesis [11,12]. 

Wnt activity is blocked by two different general strategies: dickkopfs (DKKs) and secreted frizzled-related proteins (SFRPs), which serve as decoy LRP and FZD receptors respectively [13,14]. There are five members of the SFRP family of proteins in both human and mouse genomes (SFRP1, 2, 3, 4 and 5) with SFRP1, SFRP2 and SFRP5 forming a subfamily based on sequence similarity [15]. These proteins contain a cysteine-rich domain that is homologous to the Wnt-binding domain of FZD receptor proteins [16]. However, SFRPs do not contain a transmembrane domain and therefore reside in the extracellular compartment where they antagonize Wnt signaling by binding to Wnt ligands and preventing ligand-receptor interactions and signal transduction [17]. 

SFRP family members are involved in the regulation of adipogenesis. *In vitro*, treatment with recombinant SFRP1 or SFRP2 protein in 3T3-L1 preadipocytes inhibits the anti-adipogenic Wnt/β-catenin signal and induces preadipocyte differentiation [18]. In mice, *Sfrp1* expression increases during adipogenesis and in response to HFD. In humans, elevated *SFRP1* levels are observed in mildly obese individuals and are reduced in response to extreme body weight in morbidly obese humans [18]. The role of Sfrp5 in obesity has been debated. Some groups have seen *Sfrp5* expression increase with obesity and when *Sfrp5* expression is lost there is protective effect against diet induced obesity (DIO) [18–20], while others have observed that elimination of *Sfrp5* exacerbates obesity and insulin resistance [21]. 

Increased obesity has been shown to increase pro-inflammatory cytokine production and macrophage infiltration and studies have shown the involvement of SFRP1 and Wnt5a in the inflammatory response. Specifically, Wnt5a is secreted by activated antigen presenting cells in rheumatoid arthiritis joints and promotes the production of cytokines including Interleukin (IL)-1, IL-6 and IL-8 through the Fzd5- CamKII noncononical Wnt signaling pathway [22,23]. SFRP1 has been shown to inhibit this process [24,25] and has also been shown to block leukocyte activation and cytokine production *in vitro* [26] as well as decrease neutrophil infiltration in ischemic tissue *in vivo* [27]. Thus, SFRP1 plays an important role in modulating the inflammatory response.

Considering the involvement of SFRP1 in adipogenesis and inflammation, we sought to determine the effects of diet induced obesity (DIO) in the Sfrp1^-/-^ mouse model. Our data reveal that Sfrp1^-/-^ mice exhibit increased adipocity in WAT, hepatic steatosis, glucose homeostasis irregularities and increased inflammatory repsonses. These novel findings suggest that SFRP1 is a key regulatory factor that modulates cellular responses to obesity.

## Materials and Methods

### Animals

This study was carried out in strict accordance with the recommendations in the Guide for the Care and Use of Laboratory Animals of the National Institutes of Health. The protocol was approved by the Baystate Medical Center Institutional Animal Care and Use Committee (Permit Number: 283237). Female129/C57Blk6 control mice (n=20) and129/C57Blk6 Sfrp1^-/-^ mice (n=20) were individually housed in plastic cages with food and water provided continuously, and maintained on a 12:12 light cycle. Mice (n=10/genotype) were placed on either a normal diet [(without date) Harlan Teklad global 18% protein rodent diet (#2018) containing 2.8% fat, 18.6% protein] or placed on a high fat diet [(HFD) Bio-Serv (#F1850) containing 36.0 % fat, 36.2% carbohydrate, and 20.5% protein] starting at 10 weeks of age for 12 weeks. Body weight was recorded every three days and body composition was monitored with an Echo MRI-100 (Echo Model Systems, Houston TX) prior to initiating the study, 6 weeks after putting the mice on the diet, and at the end of the study. Upon completion of metabolic measurements, mice were euthanized by CO_2_ followed by cervical dislocation and bled by cardiac puncture. Mammary glands (3^rd^, 4^th^, and 5^th^ inguinal glands), white adipose tissue [(WAT) gonadal, renal, mesenteric, and scapular fat pads], brown adipose tissue (BAT), liver, pancreas, spleen, ovary, kidney, bone, intestines and muscle were harvested, weighed and fixed in 10%buffered formalin. In addition, mammary glands, WAT, BAT, and livers were flash frozen in liquid nitrogen.

### Adipocyte size

The mean adipocyte size of the gonadal fat pad was determined by computer-assisted image analysis of adipose tissue sections stained with hematoxylin and eosin. Briefly, fluorescent images were captured at 100X (Nikon ECLIPSE TE2000-U) and measured with CellProfiler software (The Broad Institute). Cells were identified and false positives were removed by setting appropriate threshold. Images were then converted to a binary black and white image, membranes were shrunk to a skeleton (1pixel wide), and then images were inverted. Adipocytes were identified by software pixels, recorded. and converted to microns. False positive areas (area below 100um^2^) were discarded.

### Glucose tolerance test

 Glucose tolerance tests (GTTs) were carried out 2, 6, and 12 weeks after animals were placed on the ND or HFD. Briefly, mice were fasted for 16 hours, weighed, and their fasting glucose levels were measured from 1-2 μl of blood obtained from the tail vein using a glucometer (OneTouch Ultra; Lifescan, Milpitas, CA). Mice were injected with glucose (2g of D-glucose per kg of body weight) in the interperitoneal cavity and blood glucose levels were assessed 15, 30, 60, 90, and 120 minutes after injection.

### Analysis of serum insulin

 At the time of GTT analysis, blood was also collected from the tail vein into microfuge tubes before glucose injection and 15 minutes following injection. Serum was collected after centrifugation at 1,500 x g for 15 min at 4°C. Insulin levels were assessed by ultrasensitive ELISA with mouse insulin standard according to the manufacturer’s instructions (Crystal Chem Inc, Downers Grove IL). 

### RNA Isolation and Real-Time PCR analysis

Total RNA was extracted from the 5^th^ inguinal mammary gland, gonadal fat pad and liver of mice in each treatment group (n=6) as described previously [28]. Relative levels of the mRNA expression of target genes was determined by quantitative real-time PCR using the Mx3005Preal-time PCR system (Stratagene, La Jolla, CA) and all values were normalized to the amplification of β-Actin and calculated using the 2^-(ΔΔCt)^ method. The PCR primer sequences are described in Table 1 and Table S1. The assays were performed using the 1-Step Brilliant® SYBRIII® Green QRT-PCR Master Mix Kit (Stratagene) containing 200 nM forward primer, 200 nM reverse primer, and 100 ng total RNA. The conditions for cDNA synthesis and target mRNA amplification were performed as follows: 1 cycle of 50°C for 30 min;1 cycle of 95°C for 10 min; and 35cycles each of 95°C for 30 s, 55°C for 1 min, and 72°C for 30 s.

**Table 1:** PCR primer sequences for real-time PCR analysis.

*G6pc*	forward	5’- ACACCGACTACTACAGCAACAG -3’	*Chrebp-α*	forward	5’- CGACACTCACCCACCTCTTC -3’
	reverse	5’- CCTCGAAAGATAGCAAGAGTA -3’		reverse	5’- TTGTTCAGCCGGATCTTGTC -3’
*Pck1*	forward	5’-CATATGCTGATCCTGGGCATAAC-3’	*Chrebp-β*	forward	5’- TCTGCAGATCGCGTGGAG -3’
	reverse	5’- CAAACTTCATCCAGGCAATGTC - 3’		reverse	5’- CTTGTCCCGGCATAGCAAC -3’
*Slca4*	forward	5’-GACGGACACTCCATCTGTTG -3’	*Nr1h3*	forward	5’- AGGAGTGTCGACTTCGCAAA -3’
	reverse	5’-GCCACGATGGAGACATAGC -3’		reverse	5’- CTCTTCTTGCCGCTTCAGTTT -3’
*Slca2*	forward	5’- CTGGAGCCCTCTTGATGGGA -3’	*Il-6*	forward	5’-GCTACCAAACTGCATATAATCAGGA -3’
	reverse	5’-CTGGAGCCCTCTTGATGGGA -3’		reverse	5’-CCAGGTAGCTATGGTACTCCAGAA -3’
*Fasn*	forward	5’- GCTGCGGAAACTTCAGGAAAT -3’	*Tnf-α*	forward	5’-TGTCTCAGCCTCTTCTCATTCC -3’
	reverse	5’- AGAGACGTGTCACTCCTGGACTT -3’		reverse	5’-TGAGGGTCTGGGCCATAGAAC -3’
*Acly*	forward	5’-GCCCTGGAAGTGGAGAAGAT -3’	*Il-1β*	forward	5’-TGTAATGAAAGACGGCACACC -3’
	reverse	5’CCGTCCACATTCAGGATAAGA- -3’		reverse	5’-TCTTCTTTGGGTATTGTTTGG -3’
*Acaca*	forward	5’-GCGTCGGGTAGATCCGGTT -3’	*Nampt*	forward	5’- GGCAGAAGCCGAGTTCAA -3’
	reverse	5’-CTCAGTGGGGCTTAGCTCTG -3’		reverse	5’- TGGGTGGGTATTGTTTATAGTGAG -3’
*Elov16*	forward	5’- TCAGCAAAGCACCCGAAC -3’	*F4/80*	forward	5’-CTTTGGCTATGGGCTTCCAGTC -3’
	reverse	5’- AGCGACCATGTCTTTGTAGGAG -3’		reverse	5’-GCAAGGAGGACAGAGTTTATCCTG -3’
*Scd1*	forward	5’- CCCTGCGGATCTTCCTTATC -3’	*Tgf-β2*	forward	5’-TGGAGTTCAGACACTCAACACA -3’
	reverse	5’- TGTGTTTCTGAGAACTTGTGGTG -3’		reverse	5’-AAGCTTCGGGATTTATGGTGT -3’
*Srebpf1*	forward	5’- GGAGCCATGGATTGCACATT -3’	*Serpine1*	forward	5’-AGACAATGGAAGGGCAACAT -3’
	reverse	5’- GGCCCGGGAAGTCACTGT -3’		reverse	5’- AGAGACGTCTCACTCCTGGACTT-3’

### Immunohistochemistry

Immunohistochemistry (IHC) was performed on a DakoCytomation autostainer using the Envision HRP Detection system (Dako, Carpinteria, CA). Each tissue block was sectioned at 4 µm on a graded slide, deparaffinized in xylene, rehydrated in graded ethanols, and rinsed in Tris-phosphate-buffered saline (TBS). Heat induced antigen retrieval was performed in a microwave at 98°C in 0.01 M citrate buffer. After cooling for 20 minutes, sections were rinsed in TBS and subjected to primary antibodies 1:100 [Glut4 (Novus Biologicals, Littleton, CO) and F4/80 (AbD, Serotec, Hercules, CA )] for 45 minutes. Immunoreactivity was visualized by incubation with chromogen diaminobenzidine (DAB) for 5 minutes. Tissue sections were counterstained with hematoxylin, dehydrated through graded ethanols and xylene, and cover-slipped. Images were captured with an Olympus BX41 light microscope using SPOT Software 5.1 (SPOT™Imaging Solutions, Detroit, MI).

### Analysis of hepatic steatosis

Formalin fixed livers were paraffin-embedded and sections were cut on a Leica microtome at a thickness of 4 m on Superfrost-plus slides, and stained with hematoxylin and eosin (H&E) per standard protocol. Frozen sections were used for the detection of lipid. Freshly dissected liver was cryoprotected in 30% sucrose overnight and then flash frozen in Tissue-Tek OCT compound (Sakura). Cryosections (10 μm thick) were processed for Oil Red O staining in a 0.3% solution of Oil Red O (Sigma, St. Louis, MO) in 60% isopropanol for 15 min. After washes in 60% isopropanol, sections were counterstained with Mayer’s hematoxylin. Images were captured with an Olympus BX41 light microscope using SPOT Software 5.1 (SPOT™Imaging Solutions).

### Statistical Analysis

Results were analyzed using a two-way ANOVA with *Sfrp1* loss and HFD treatment as the main effects unless otherwise stated. *Post hoc* tests, where appropriate, were performed by Bonferroni’s *t* test. Bonferroni’s *t* test uses the mean square error from the ANOVA table as a point estimate of the pooled variance (Graphpad Prism, San Diego, CA). Group means were compared using Student’s t-tests (Graphpad Prism) and results with P<0.05 were considered significant. Multilevel mixed-effects linear regression was used to compare genotype and diet groups on mean change in insulin secretion from 0-15 minutes, accounting for any within-subject correlation of repeated measurements of mice at 2, 6 and 12 weeks. Grubb’s test was used on all data to identify statistical outliers (http://www.graphpad.com/quickcalcs). Statistical outliers were identified in some data sets, but the overall results were not altered by omission. A few samples were lost during processes; therefore, there are some unequal sample sizes.

### Supporting Information

 The experimental procedures utilized for generation of supplemental figures can be found in File S1.

## Results

### Loss of Sfrp1 exacerbates weight gain and adiposity in mice fed a HFD

**Figure 1:**
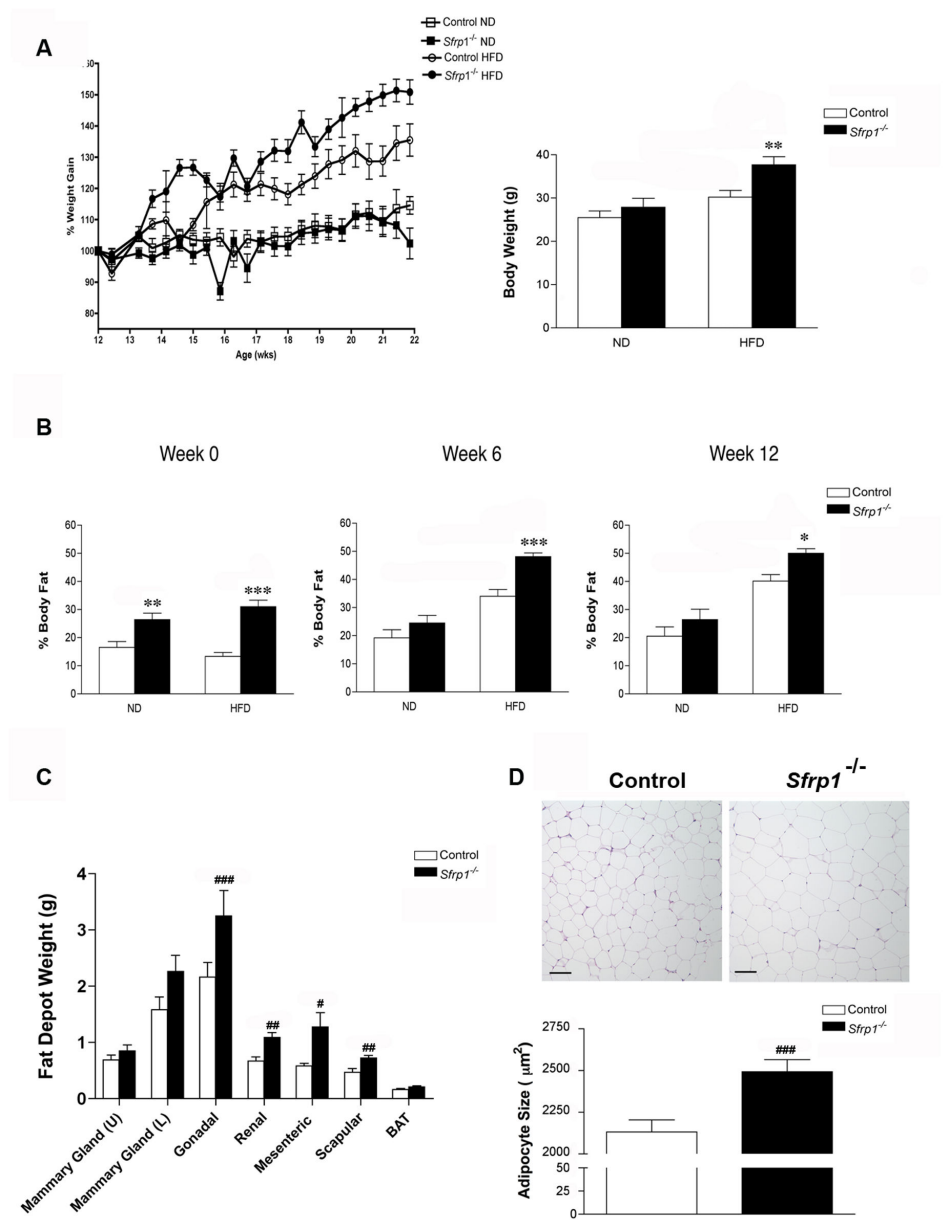
Effects of high-fat diet-induced obesity on weight gain and adiposity. (A) *Left* *panel*, The body weight of control and Sfrp1^-/-^ mice put on a ND and HFD was monitored every 3 days after being put on the diet and changes are displayed graphically as percent initial body weight. *Right* *panel*, Final body weight after 12 weeks on the diet. (B) Body composition was analyzed by NMR-MRI at the start of the diet (0 weeks), mid-study (6 weeks), and at the end of the study (12 weeks) and fat mass was normalized to body weight and expressed as percent body fat. (C) Average weight of upper mammary glands (3^rd^ & 4^th^ inguinal), lower mammary gland (5^th^ inguinal), gonadal fat pad, renal fat pad, mesenteric fat pad, scapular fat pad, and brown adipose tissue harvested from mice fed a HFD for 12 weeks. (D) *Upper* *panel*, Representative images of H&E staining of gonadal fad pads dissected from mice fed a HFD for 12 weeks. *Lower* *panel*, Quantification of gonadal adipocyte size. (*p<0.05,**p<0.01, ***p<0.001 significantly different from control mice fed a ND using Bonferroni’s *t* test after a two-way ANOVA; ^#^p<0.05, ^##^p<0.01, ^###^p<0.001 significantly different from control mice fed a HFD using student’s *t* test.) .

Since it has previously been shown that *Sfrp1* levels are reduced in the adipose tissue of severely obese mice and humans [18], we chose to evaluate the effects of diet-induced obesity (DIO) in Sfrp1^-/-^ mice. Control and Sfrp1^-/-^ mice were fed either a high fat diet (HFD) or a normal diet (ND) for 12 weeks after reaching 10 weeks of age. Both control and Sfrp1^-/-^ mice fed a HFD gained weight as expected (Figure 1A, left panel), with a more rapid increase in Sfrp1^-/-^ mice when compared to control mice. A two-way ANOVA revealed a significant effect on final body weight in response to *Sfrp1* loss (F_1,36_=7.95; *P*<0.01) and the HFD (F_1,36_=17.04; *P*<0.001), but there was no interaction among these two main effects (F_1,36_=2.07; *P*>0.05) (Figure 1A, right panel). Body fat analysis demonstrated that body fat percentage is affected by *Sfrp1* loss (F_1,36_=45.25; *P*<0.0001) at the time the study was initiated, but there is understandably no diet effect (F_1,36_=0.12; *P*>0.05) or interaction between the two main effects (F_1,36_=3.67; *P*>0.05) (Figure 1B, 0 Weeks). On the other hand, at the later time points, body fat percentage was affected by both *Sfrp1* loss (6 weeks: F_1,35_=16.05; *P*<0.001; 12 weeks: F_1,35_=8.03; *P*<0.01) and the HFD (6 weeks: F_1,35_=63.83; *P*<0.0001; 12 weeks: F_1,35_=59.31; *P*<0.0001), but there was no interaction between the two main effects (6 weeks: F_1,35_=3.37; *P*>0.05; 12 weeks: F_1,35_=0.51; *P*>0.05) (Figure 1B, 6 &12 weeks). Evaluation of fat depot weight at the end of the study demonstrated that in response to a HFD, there was a significant increase in the white adipose tissue (WAT) from Sfrp1^-/-^ mice, both the visceral gonadal fat pad and in the subcutaneous scapular fat pad (Figure 1C). Loss of *Sfrp1* did not affect the weight of 3^rd^, 4^th^, and 5^th^ inguinal mammary glands and also did not change the weight of brown adipose tissue (BAT) (Figure 1C). Additionally, organ weight was recorded in animals on both diets and there were no significant changes in response to *Sfrp*1 deficiency (Figure S1). Measurement of the adipocytes comprising the gonadal fat pad demonstrated that there is a significant increase in adipocyte size from Sfrp1^-/-^ mice compared to control mice fed a HFD (Figure 1D). Taken together these data illustrate that loss of *Sfrp1* exacerbates weight gain and adiposity in response to HFD. 

### Glucose clearance and insulin secretion is dysregulated in Sfrp1^-/-^ mice in response to DIO

**Figure 2:**
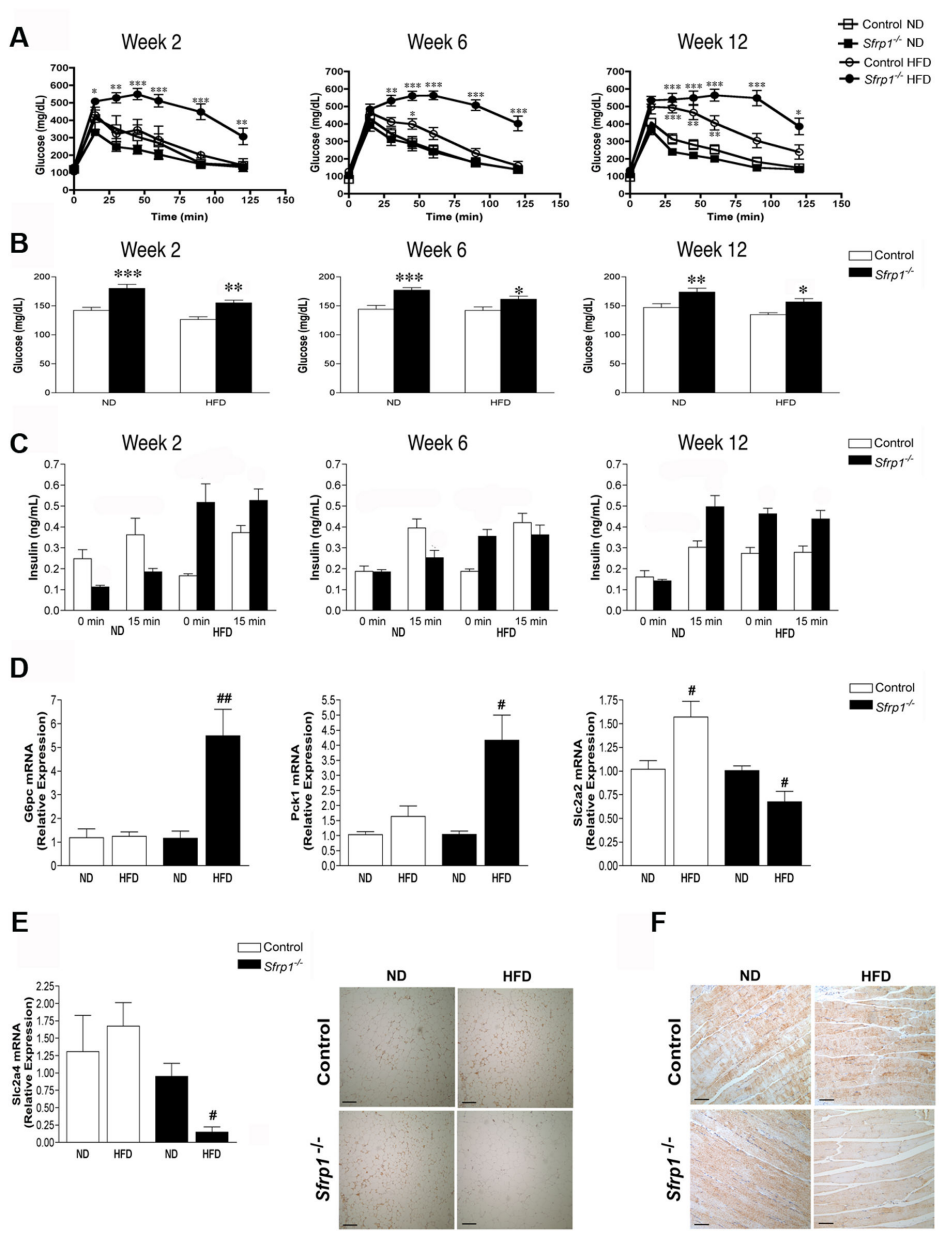
Aberrant glucose homeostasis and insulin secretion observed in Sfrp1^-/-^ mice. (A) Glucose tolerance test (GTT) was performed at three separate time points during the study (2 weeks, 6 weeks, and 12 weeks). After a 16-18 h fast, mice were injected with 2g/kg BW glucose and blood glucose levels were monitored for 2 h. (B) Blood glucose levels were measured prior to GTT initiation. (C) Plasma insulin levels were measured before (0 min) and after (15 min) glucose injection. (D) For real-time PCR analysis of *G6pc*, *Pck*1, and *Slca2* gene expression, total RNA was isolated from liver of mice in each treatment group (n=6). The results shown represent experiments performed in duplicate and normalized to the amplification of β-actin mRNA. Bars represent mean ± SEM of the relative expression with respect to ND fed mice. (E) *Left* *panel*, For real-time PCR analysis of *Slca4* gene expression, total RNA was isolated from the gonadal fad pad from mice in each treatment group (n=6). Real-time PCR analysis was carried out as described above. *Right* *panel*, gonadal fat pad sections were subjected to immunohistochemical analysis and stained for *Slc2a4* (brown chromogen).Representative images were captured at 100X and are displayed for mice in each treatment group (scale bar 200 μm). (F) Muscle sections were stained for *Slc2a4* and 200X images were captured as described above (scale bar 100 μm). (*p<0.05,**p<0.01, ***p<0.001 significantly different from control mice fed a ND using Bonferroni’s *t* test after a two-way ANOVA; ^#^p<0.05, ^##^p<0.01, ^###^p<0.001 significantly different from respective ND fed mice using student’s *t* test.) .

To explore glucose homeostasis and insulin release, a glucose tolerance test (GTT) was performed at 3 different time points within the study (Figure 2A). At all three time points (2, 6, and 12 weeks), Sfrp1^-/-^ mice on a HFD showed significantly delayed glucose clearance in response to a glucose bolus compared to all other groups (Figure 2A). Fasting glucose was measured at the beginning of each GTT time point and a two-way ANOVA revealed that at all three time points, there was a significant effect on fasting blood glucose levels in response to both *Sfrp1* loss (2 weeks: F_1,35_=36.16; *P*<0.0001; 6 weeks F_1,35_=21.67; *P*<0.0001; 12 weeks: F_1,35_=18.5; *P*<0.0001) and at 2 weeks and 12 weeks, there was an effect in response to the HFD (2 weeks: F_1,35_=13.67; *P*<0.0001; 6 weeks F_1,35_=2.47; *P*>0.05; 12 weeks: F_1,35_=6.73; *P*<0.05). However, there were no interactions between the main effects (2 weeks: F_1,35_=0.75; 6 weeks: F_1,35_=1.38, *P*<0.05; 12 weeks: F_1,35_=0.689; *P*<0.05) (Figure 2B). 

We next measured insulin secretion via ELISA throughout the study (Figure 2C & Table 2). Table 2 shows regression-adjusted mean change in insulin secretion levels from 0 - 15 minutes according to diet and genotype. Mean change in insulin secretion was significantly higher in mice fed a ND compared to mice fed a HFD after adjusting for genotype (p< 0.001). Likewise, mean change in insulin secretion was significantly higher in control mice compared to Sfrp1^-/-^ mice after adjusting for diet (p<0.01). Interestingly, there was also a significant interaction between the diet and genotype on insulin change. The effect of *Sfrp1* loss on lowering the change in insulin was present only in mice fed a HFD. In mice fed a ND, there was no difference in insulin change between control and Sfrp1^-/-^ mice (Table 2). This hyperinsulinemic response in Sfrp1^-/-^ mice was mimicked *ex vivo* in pancreatic islets. Specifically, a two-way ANOVA revealed that there was a significant effect on insulin secretion in response to both *Sfrp1* loss (F_1,8_=12.13; *P*<0.01) as well as glucose stimulation (F_1,8_=32.43; *P*<0.001), and there was also an interaction between these main effects (F_1,8_=9.99; *P*<0.05). (Figure S2A). Interestingly, the HFD did not affect the quantity of glucagon expressing alpha cells in pancreatic islets (Figure S2B). The total number of islets/slide was affected by *Sfrp1* loss (F_1,19_=4.44; *P*<0.05), but not the HFD (F_1,19_=4.44; *P*<0.05), and there was a significant interaction between the two main effects (F_1,19_=7.72; *P*<0.01), (Figure S2C). Hyperglycemia in the presence of high insulin might also suggest either enhanced insulin tolerance or a secretion of inactive insulin. An insulin tolerance test (ITT) suggested that Sfrp1^-/-^ mice may not be systemically insulin resistant and when they finally do exhibit resistance by this test, it is at a rate similar to the control animals on a high fat diet (Figure S2E). Finally, immunohistochemical detection of C-peptide in pancreatic islets confirmed that the insulin secreted by Sfrp1^-/-^ mice on a HFD is indeed cleaved and active (Figure S2D). 

**Table 2:** Regression-adjusted mean change in insulin secretion from 0-15 minutes.

	***Mean Change***	***Std. Err.***
***Diet***		
HFD	0.078 ng/mL	0.0228057 ng/mL
ND	0.159 ng/mL ***	0.0232096 ng/mL
***Genotype***		
*Sfrp1-/-*	0.0809 ng/mL	0.0232096 ng/mL
Control	0.154 ng/mL**	0.0228057 ng/mL
***Diet/Genotype***		
HFD/*Sfrp1*-/-	0.007ng/mL**	0.0328183 ng/mL
HFD/Control	0.147 ng/mL	0.0317055 ng/mL
ND/*Sfrp1*-/-	0.156 ng/mL	0.0328183 ng/mL
ND/Control	0.161 ng/mL	0.0328183 ng/mL

**p<0.01,*** p<0.001

### DIO misregulates the expression of regulatory gluconeogenesis enzymes and glucose transporters in Sfrp1^-/-^ mice

Glucose is internally produced in the liver by way of gluconeogenesis. Wnt signaling regulates gluconeogenesis by controlling the expression of glucose-6-phosphatase (*G6pc*), and the rate limiting enzyme, PEP carboxykinase (*Pck1*) [29]. Therefore, we measured the mRNA levels of *G6pc* and *Pck1* in the livers of our animals (Figure 2D). We observed an aberrant response in Sfrp1^-/-^ mice fed a HFD in that despite the elevated glucose levels in these animals, they exhibited a significant increase in the expression of both *G6pc* and *Pck1* compared with Sfrp1^-/-^ mice fed a ND. Canonical Wnt signaling has been shown to upregulate the expression of these enzymes [30] and real-time PCR analysis of several Wnt ligand transcripts revealed that the Wnt3a ligand is significantly elevated in response to a HFD in Sfrp1^-/-^ mice (Figure S3B). 

Glucose clearance is due to several glucose transporters in the muscle, liver and fat tissues, which carry the circulating glucose from the blood stream into cells for energetic storage and so we examined the expression of glucose transporter 2 (Slc2a2 *aka* Glut2) in the liver. As expected, control mice fed a HFD expressed elevated levels of *Slca2* when compared with control mice fed a ND. However, the expression of *Slca2a2* was significantly reduced in Sfrp1^-/-^ mice fed a HFD compared to ND fed mice. Similarly, in the gonadal fat pad, the transcript and protein levels of glucose transporter 4 (Slc2a4 *aka* Glut4) were significantly lower in Sfrp1^-/-^ mice fed a HFD (Figure 2E). Glucose transporter 4 is responsible for insulin stimulated glucose transport into the muscle and immunohistochemical analysis revealed that HFD fed Sfrp1^-/-^ mice express reduced Slc2a4 levels in muscle tissue (Figure 2F). These results suggest that *Sfrp1* loss results in a multifactorial effect, which includes misregulated gluconeogenesis, with a slightly compromised ability to clear glucose, resulting in impaired glucose homeostasis and hyperinsulinemia.

### Sfrp1^-/-^ mice fed a HFD exhibit hepatic steatosis

**Figure 3:**
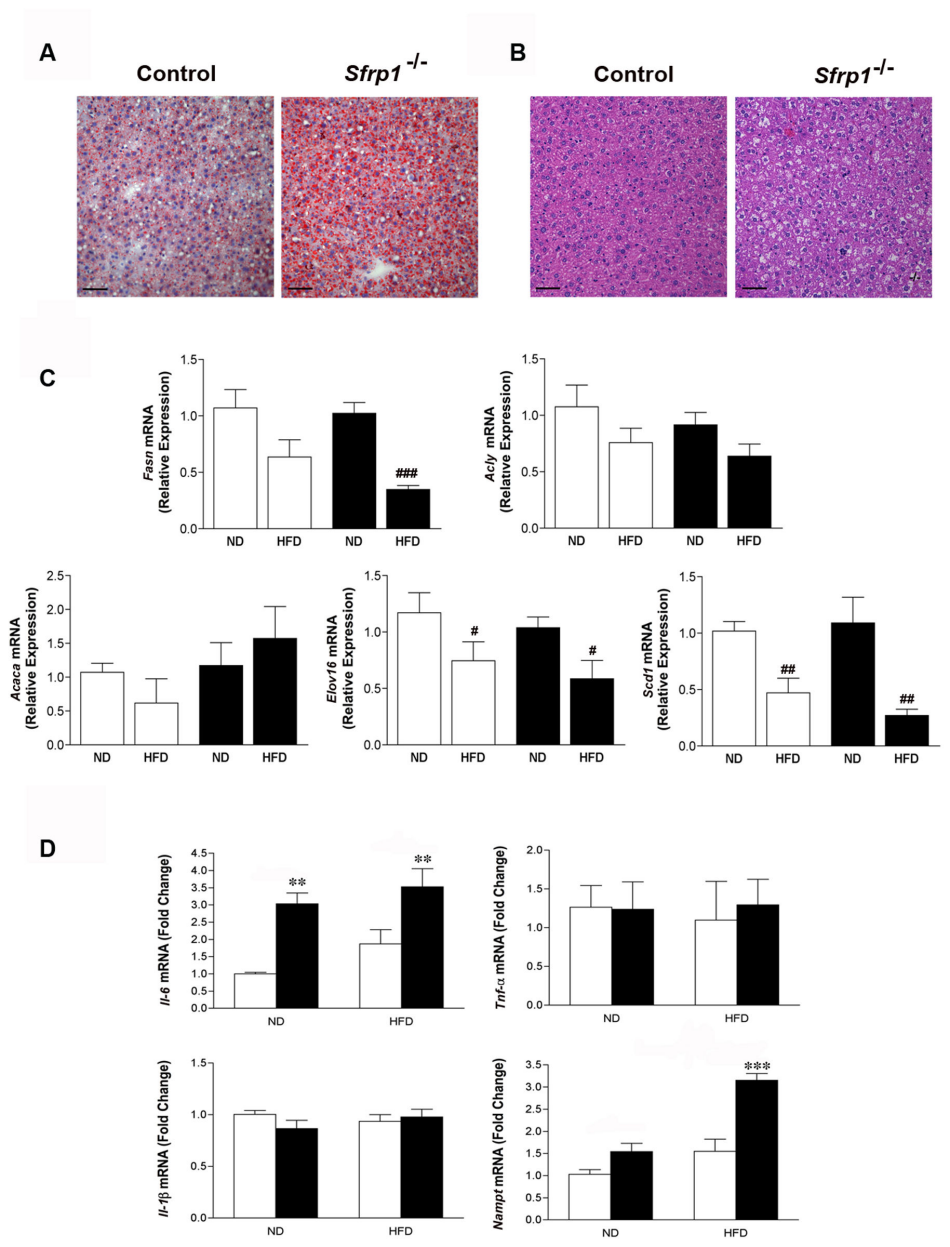
Hepatic steatosis and inflammation exhibited by Sfrp1^-/-^ mice. (A) Steatosis is distinguished by Oil Red O staining of lipids. Images were captured at 400X and represent control mice and Sfrp1^-/-^ mice on a HFD (scale bar 50 μm). (B) H&E staining of livers and captured as described above. (C) Total RNA from the livers in each group (n=6) was utilized for real-time PCR analysis of *Fasn*, Acly, and *Acaca, Elov*16, and *Scd*1 gene expression. The results shown represent experiments performed in duplicate and normalized to the amplification of β-actin mRNA. Bars represent mean ± SEM of the relative expression with respect to ND fed mice. (D) Real-time PCR analysis of liver Il-6*, Tnf*-α, Il-1β, and *Nampt* gene expression was carried out as described above. Bars represent mean ± SEM of the difference in fold change compared with control ND fed mice. (*p<0.05, **p<0.01, ***p<0.001 significantly different from control mice fed a ND using Bonferroni’s *t* test after a two-way ANOVA; ^#^p<0.05, ^##^p<0.01, ^###^p<0.001 significantly different from respective ND fed mice using student’s *t* test.).

Considering that we observed significant *Sfrp1* dependent changes in the gluconeogenic transcripts in the liver, and non-alcoholic fatty liver disease is tightly associated with obesity and hyperglycemia [31], we chose to examine the effects of a HFD in the livers of these mice. Although we did not observe any significant differences in gross liver weight (Figure S1), we did observe increased liver steatosis in Sfrp1^-/-^ mice fed a HFD. This is clearly illustrated by oil red O staining of frozen liver sections, which have noticeably more lipid accumulation compared to control HFD fed mice (Figure 3A). These findings support the notion that the observed hepatic steatosis is a direct result of the HFD on other tissues rather than an effect of *Sfrp1* loss on the misregulation of lipogenesis in the liver. 

### Sfrp1 depletion increases the expression of hepatic pro-inflammatory cytokines

The accumulation of fat in the liver is also accompanied byprogressive inflammation of the liver (steatohepatitis). Thus, we looked at the effect of DIO on inflammation in our animal models. No histological signs of inflammatory infiltrate were observed in any of the livers regardless of treatment group (data not shown). A two-way ANOVA revealed that there were no effects on *Tnf-*α or Il-1β mRNA expression in response to *Sfrp1* loss (*Tnf-*α: F_1,20_=0.8272; P>0.05; Il-1β: F_1,20_=0.51; P>0.05) or the HFD (*Tnf-*α: F_1,20_=0.8841 P>0.05; Il-1β: F_1,20_=0.12; P>0.05). There was also no interaction between these two main effects (*Tnf-*α: F_1,20_=0.09 P>0.05; Il-1β: F_1,20_=1.83; P>0.05). However, a two-way ANOVA showed that *Il-6* mRNA expression levels were significantly affected by *Sfrp1* loss (F_1,20_=25.01; P<0.0001), though there was no effect in response to the HFD (F_1,20_=3.44; P>0.05) and there was no interaction between the main effects (F_1,20_=0.25; P>0.05). We next measured the mRNA levels of the pro-inflammatory adipokine Visfatin (*Nampt*), as it potently induces the production of *Il-6* [32]. A two-way ANOVA revealed a significant effect on *Nampt* expression in response to *Sfrp1* loss (F_1,20_=31.78; P<0.0001) and the HFD (F_1,20_=31.86; P<0.0001), as well as a significant interaction between these two main effects (F_1,20_=8.34; P<0.01) (Figure 3D). 

### Loss of Sfrp1 increases de novo lipogenesis in the gonadal fat pad

**Figure 4:**
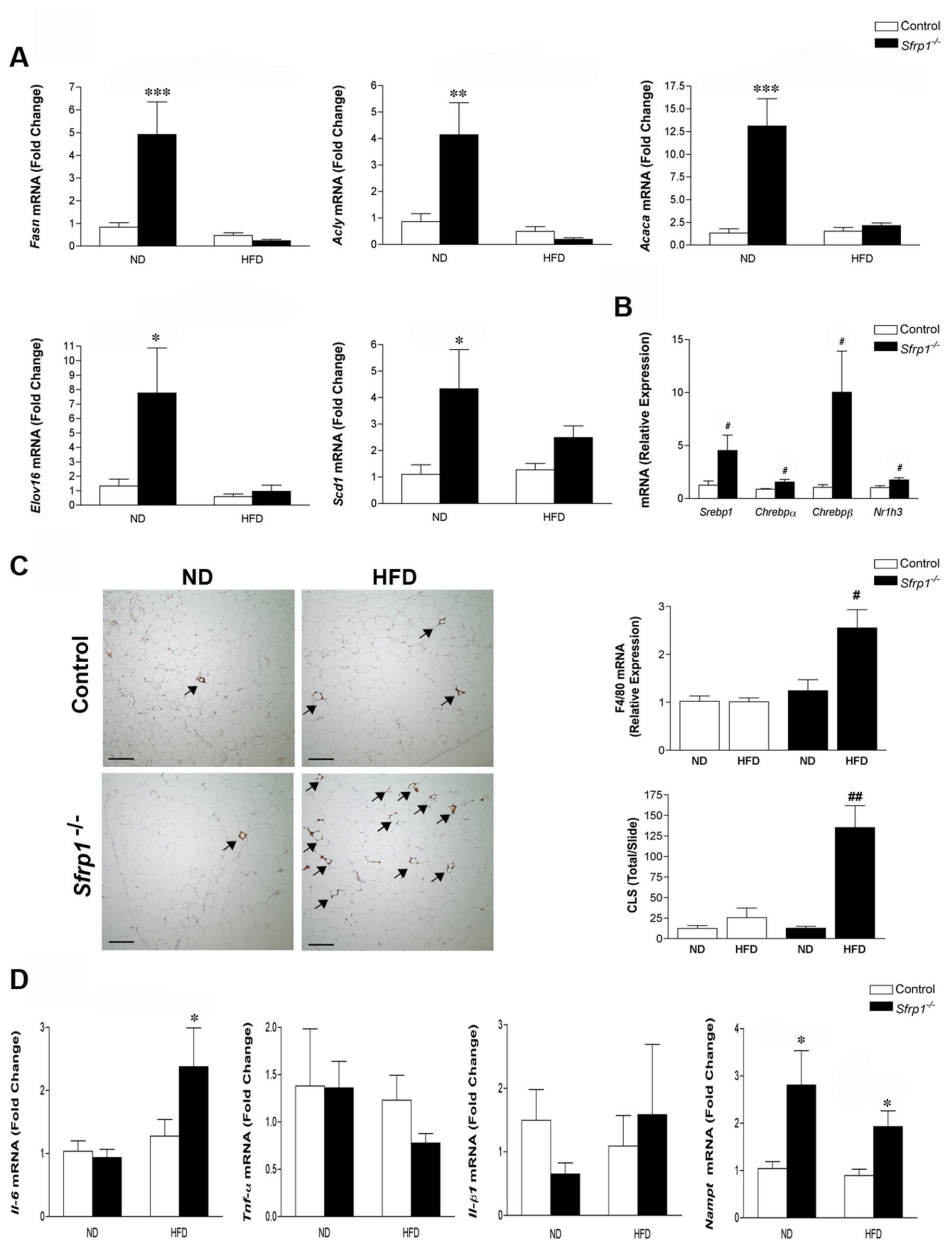
Analysis of de novo lipid synthesis and the inflammatory signature in the gonadal fat pad. (A) Total RNA from the gonadal fat pad in each group (n=6) was utilized for real-time PCR analysis of *Fasn*, Acly, and *Acaca, Elov*16, and *Scd*1 gene expression. The results shown represent experiments performed in duplicate and normalized to the amplification of β-actin mRNA. Bars represent mean ± SEM of the difference in fold change compared with control ND fed mice. (B) Real-time PCR was carried out utilizing RNA from the gonadal fat pad of control mice and Sfrp1^-/-^ mice on a ND to measure the relative mRNA expression of the following transcription factors: *Srebp1, Chrebp-α*, *Chrebp*-β, and *Nr1h3*. The experiments were carried out as described above and bars represent mean ± SEM of the difference in fold change compared with control ND fed mice. (C) *Left* *panel*, gonadal fat pad sections were subjected to immunohistochemical analysis, stained for F4/80 (brown chromogen), and representative images were captured at 100X and are displayed for mice in each treatment group (scale bar 200 μm). *Right* *upper* *panel*, gondal fat pad RNA was used for real-time PCR analysis of F4/80 mRNA, experiments were carried out as described, and bars represent mean ± SEM of the relative expression with respect to ND fed mice. *Right* *lower* *panel*, The total number of CLSs (see arrow inset) were counted in F4/80 stained gonadal fat pad sections (n=4/genotype) (D) Real-time PCR analysis of liver Il-6*, Tnf*-α, Il-1β, and *Nampt* gene expression was carried out as described above. Bars represent mean ± SEM of the difference in fold change compared with control ND fed mice. (*p<0.05, **p<0.01, ***p<0.001 significantly different from control mice fed a ND using Bonferroni’s *t* test after a two-way ANOVA; ^#^p<0.05, ^##^p<0.01, significantly different from respective ND fed mice using student’s *t* test.).

We demonstrate that mice deficient in *Sfrp1* exhibit an increase in visceral fat pad weight, with the gonadal fat pad showing the highest significance (Figure 1C). Therefore, to elucidate the contributing factors leading to the increased adiposity, we took a closer look at the gonadal fat pad *de novo* lipogenesis gene expression profile. Real-time PCR analysis revealed that the mRNA transcript levels of several *de novo* lipogenesis genes (*Acaca, Acly, Fasn, , Elov16*, and *Scd1*) were affected by loss of *Sfrp1*. Specifically, a two-way ANOVA revealed that there was a significant effect on *Acaca* expression in response to *Sfrp1* loss (F_1,17_=27.30; P<0.0001) and the HFD (F_1,17_=20.46; P<0.001), as well as a significant interaction between these two main effects (F_1,17_=22.14; P<0.001). Similarly, *Acly* expression was affected in response to *Sfrp1* loss (F_1,15_=7.72; P<0.05) as well as the HFD (F_1,15_=16.20; P<0.01), and there was a significant interaction between these two main effects (F_1,15_=11.13; P<0.01). *Fasn* expression was also altered in response to *Sfrp1* loss (F_1,16_=11.01; P<0.01) as well as the HFD (F_1,16_=19.04; P<0.001), and there was a significant interaction between these two main effects (F_1,16_=13.92; P<0.01). *Elov16* mRNA levels were affected by *Sfrp1* loss (F_1,18_=5.53; P<0.05) as well as the HFD (F_1,18_=6.77; P<0.05) and there was a significant interaction between these two main effects (F_1,18_=4.41; P<0.05). Lastly, *Scd1* expression was affected by *Sfrp1* loss (F_1,18_=6.42; P<0.05), though there was no effect in response to the HFD (F_1,18_=0.90; P>0.05) and there was no interaction between the main effects (F_1,18_=1.31; P>0.05) (Figure 4A). As expected, when the Sfrp1^-/-^ mice were fed a HFD and insulin levels increased, the mRNA expression of these genes was reduced to basal levels, with the exception of *Scd1* (Figure 4A). 

 We next sought to determine whether *Sfrp1* loss affects the transcription factors involved in regulating the mRNA expression *de novo* lipogenesis genes. These transcription factors control the *de novo* lipogenesis process in response to either glucose levels (Chrebp-α, Chrebp-β) or insulin (Srebp1). The expression of each of these genes is significantly elevated in the gonadal fat pad of Sfrp1^-/-^ mice on a ND (Figure 4B). The Lxrα transcription factor (Nr1h3) is known to regulate cholesterol homeostasis and uptake, and our results demonstrate that there is a significant increase in expression of *Nr1h3* mRNA in ND fed Sfrp1^-/-^ mice (Figure 4B). Interestingly, canonical Wnt signaling is enhanced in response to *Fasn* [33] and analysis of Wnt ligands in the gonadal fat pad shows that *Wnt3a* is overexpressed in Sfrp1^-/-^ mice (Figure S4). 

### Sfrp1^-/-^ mice fed a HFD exhibit enhanced macrophage infiltration and cytokine production in the gonadal fat pad

Since visceral fat is prone to inflammation and Wnts have been shown to augment macrophage cytokine production [25], we investigated the inflammatory state of the gonadal fat pad. We first looked at the expression of F4/80 (macrophage marker) in control and Sfrp1^-/-^ mice in response to a HFD and found that Sfrp1^-/-^ mice exhibit a significant increase in F4/80 mRNA (Figure 4C, right panel). The F4/80 protein is a 160 kDa cell surface glycoprotein and under lipolytic conditions, F4/80 positive macrophages cluster around dying adipocytes and are referred to as Crown-like structures (CLS). These structures are frequently detected in fat tissue of obese patients and are associated with inflammation [34,35]. Our images reveal that more CLSs are present in Sfrp1^-/-^ mice fed a HFD (Figure 4C, left panel) and quantification confirmed that these observations were significantly different from control animals on a HFD (Figure 4C, right panel). We then looked at the gene expression of pro-inflammatory cytokines (Il-6, *Tnf*-α, Il-1β and *Nampt*). A two-way ANOVA revealed that Il-6 mRNA expression is not affected by *Sfrp1* loss (F_1,13_=3.26; P>0.05) but is significantly affected by the HFD (F_1,13_=9.23; P<0.01) and there is a significant interaction between these two main effects (F_1,13_=4.72; P<0.05). The expression of *Nampt* is affected by *Sfrp1* loss (F_1,17_=9.42; P<0.01) but it is not significantly affected by the HFD (F_1,17_=1.26; P>0.05) and there is no significant interaction between these two main effects (F_1,17_=0.65; P>0.05). Although *Tnf-*α was not affected in response to *Sfrp1* loss (F_1,18_=0.82; P>0.05) or in response to the HFD (F_1,18_=1.04; P>0.05), there is a significant interaction between these two main effects (F_1,18_=4.48; P<0.05). The expression of Il-1β was not affected by *Sfrp1* loss (F_1,20_=0.51 P>0.05) or the HFD (F_1,20_=0.12; P>0.05) and there was no interaction between the main effects (F_1,20_=1.83; P>0.05).

### Sfrp1^-/-^ mice fed a HFD exhibit enhanced macrophage infiltration and altered cytokine expression in the mammary gland

**Figure 5:**
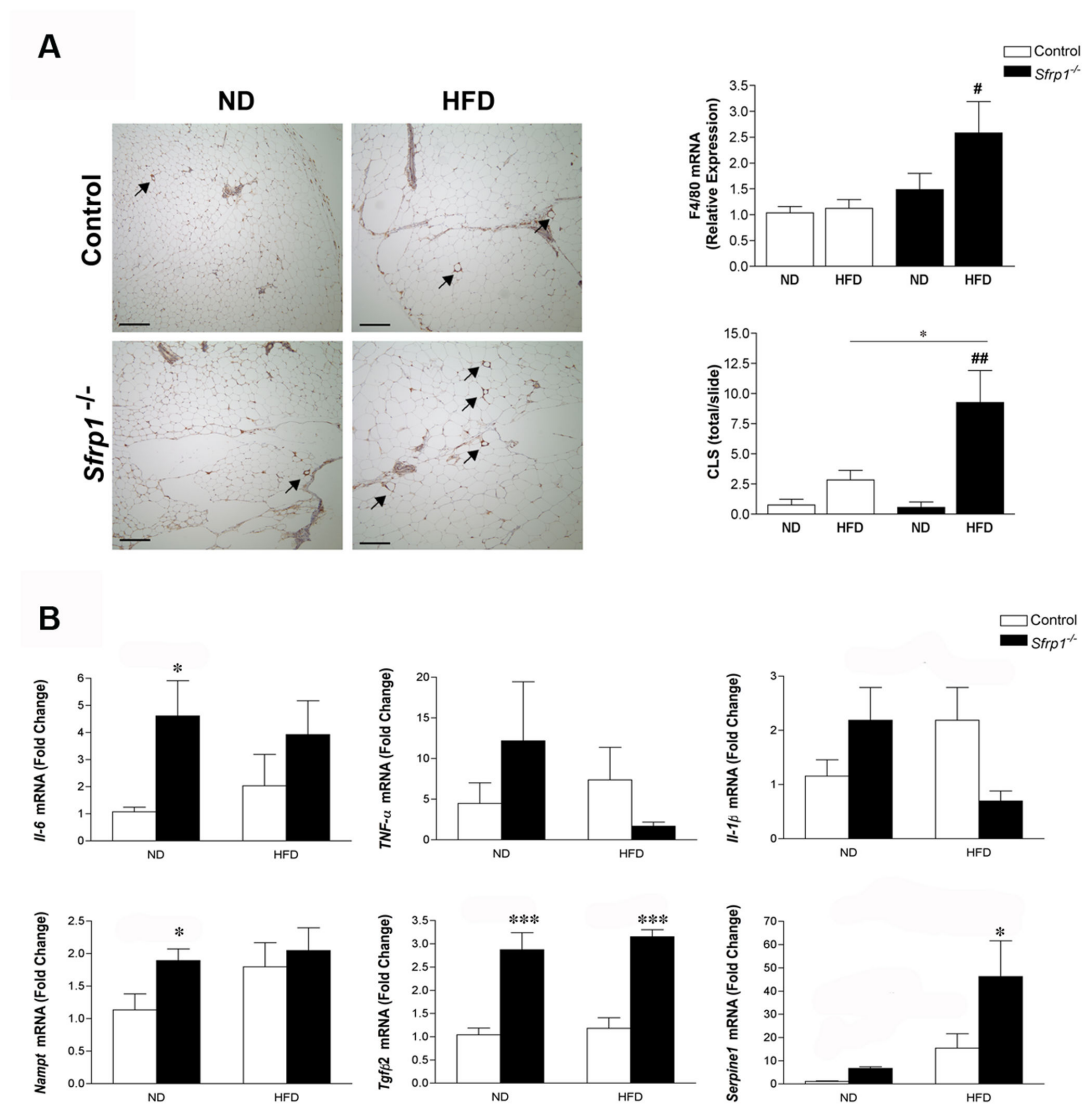
Macrophage infiltration and cytokine expression in the mammary gland. *Left* *panel*, 3^rd^ & 4^th^ inguinal mammary gland sections were subjected to immunohistochemical analysis, stained for F4/80 (brown chromogen), and representative images were captured at 100X are displayed for mice in each treatment group (scale bar 200 μm). *Right* *upper* *panel*, total RNA was harvested from 5^th^ inguinal mammary glands and employed for real-time PCR analysis of F4/80 mRNA, experiments were carried out as described, and bars represent mean ± SEM of the relative expression with respect to ND fed mice. *Right* *lower* *panel*, The total number of CLSs (see arrow inset) were counted in F4/80 stained mammary gland sections (n=4/treatment group) (D) Real-time PCR analysis of liver Il-6*, Tnf*-α, Il-1β, *Nampt, Tgf*-β2, and *Serpine*1 gene expression was carried out as described above. Bars represent mean ± SEM of the difference in fold change compared with control ND fed mice. (*p<0.05, ***p<0.001 significantly different from control mice fed a ND using Bonferroni’s *t* test after a two-way ANOVA; ^#^p<0.05, ^##^p<0.01, significantly different from respective ND fed mice using student’s *t* test.).

It has previously been shown that loss of SFRP1 alters the growth and behavior of mammary epithelial cells, *in vitro* and *in vivo*, in such a manner that they exhibit characteristics of breast cancer cells [28,36]. Together with the notion that obesity is associated with cancer risk, due in part to inflammation, and detection of increased macrophage infiltration in the gonadal fat pad from Sfrp1^-/-^ mice fed a HFD mice, we investigated the inflammatory state of the mammary fat pad. Real-time PCR analysis revealed that there is a significant increase in F4/80 expression in Sfrp1^-/-^ mice fed a HFD diet (Figure 5A, left panel). Consistent with these findings, mRNA levels of CD68 (another macrophage marker) mirror those of F4/80 expression (Figure S5). As expected, immunohistochemical analysis of F4/80 demonstrated increased macrophage infiltration in Sfrp1^-/-^ mice fed a HFD (Figure 5A, right panel). CLSs in the mammary gland of Sfrp1^-/-^ mice fed a HFD were significantly increased compared to other groups, regardless of diet or genotype, confirming the increased macrophage activity. 

We next explored the expression of inflammatory cytokines in the mammary gland fat pad and found that the expression of pro-inflammatory cytokine genes was variable (Figure 5B). A two-way ANOVA demonstrated that the expression of Il-6 was affected in response to *Sfrp1* loss (F_1,20_=6.42; P<0.05) but was not significantly affected by the HFD (F_1,20_=0.02; P>0.05) and there was no significant interaction between these two main effects (F_1,20_=0.59; P>0.05). The mRNA levels of *Tnf-*α were not altered in response to *Sfrp1* loss (F_1,18_=0.05; P>0.05) or by the HFD (F_1,18_=0.69; P>0.05) and there was not a significant interaction between these two main effects (F_1,18_=2.14; P>0.05). Although the expression of Il-1β was not affected in response to *Sfrp1* loss (F_1,20_=0.24; P>0.05) or by the HFD (F_1,20_=0.24; P>0.05), there was significant interaction between these two main effects (F_1,20_=7.39; P<0.05). Similar to our findings in the liver and the gonadal fat pad, there was a significant effect on *Nampt* mRNA levels in response to *Sfrp1* loss (F_1,20_=4.49; P<0.05) but there is no effect induced by the HFD (F_1,20_=0.85; P>0.05) and there was no significant interaction between these two main effects (F_1,_=1.69; P>0.05). 

To elucidate the rationale for the observed increase in macrophage infiltration, we chose to look at the expression of *Tgf*-β2 because it is a potent macrophage chemoattractant. Previous research has demonstrated that loss of S*frp1* promotes the expression of *Tgf*-β2 in mammary epithelial cell lines and in the murine mammary gland [36,37]. A two-way ANOVA showed that there was an effect on *Tgf*-β2 expression in response to *Sfrp1* loss (F_1,18_=77.5; P<0.0001) but there was no effect in response to the HFD (F_1,18_=0.94; P>0.05) and there was no significant interaction between these two main effects (F_1,18_=6.12; P>0.05). Furthermore, a well known downstream target of *Tgf*-β2 signaling and biomarker for cancer risk, Plasminogen Activator-1 (*Serpine*1) was significantly affected by *Sfrp1* loss (F_1,19_=4.35; P<0.05) and the HFD (F_1,19_=9.55; P<0.01), but there was no interaction between these two main effects (F_1,19_=2.09; P>0.05). These results suggest that there is an altered immune signature in the mammary environment that may be signaling to the epithelium.

## Discussion

The present study demonstrates that loss of *Sfrp1* exacerbates adiposity, weight gain, glucose homeostasis and inflammation in mice on a HFD. It is well established that Wnt signaling is important for preadipocyte differentiation and adipogenesis [2]. Some ligands act as inhibitors, such as Wnt10a and Wnt10b [8,9], and other ligands promote adipogenesis, such as Wnt5b and Wnt4 [11,12] in both a canonical and non-canonical fashion. We looked at the expression of Wnt ligands in the gonadal fat pad and found that there were no differences in the expression of *Wnt5b* or *Wnt10b* (Figure S4). However, *Sfrp1* loss did induce an increase in *Wnt3a* expression regardless of diet (Figure S4). Lagathu et. al. have previously shown that the *Sfrp1* expression profile in mice and humans exhibits a bell-shaped pattern in response to a HFD and a decrease in Wnt/β-catenin signaling [18]. Since we observed an increase in *Wnt3a* expression in the gonadal fat pad, we hypothesized that canonical Wnt/β-catenin signaling would be increased in Sfrp1^-/-^ mice, but no differences were detected in the gonadal or liver tissue of hallmark canonical target transcripts including c-Myc*, Ccdn1* and *Axin2* (data not shown). However, given the body of literature linking Wnt signaling to breast cancer [38], we measured the expression of *Axin2* in mammary tissue of control and Sfrp1^-/-^ mice fed a ND or HFD because it is a direct readout of Wnt signaling. We found that contrary to the gonadal fat pad, Sfrp1^-/-^ mice fed a HFD exhibit an up-regulation of *Axin2* expression in the mammary fat pad (Figure S6). Collectively, some of the the observed effects in response to *Sfrp1* loss must be independent of Wnt signaling since SFRP1 affects a myriad of cell signaling pathways such as regulating thrombospondin, ADAM proteases and RANKL [37,39–42]

To determine the mechanism by which Sfrp1^-/-^ mice exhibit enhanced growth of WAT fat depots, we analyzed genes involved in *de novo* lipogenesis. Lxr-α, Srebp1 and Chrebp are three transcription factors critical for regulating a majority of target genes that comprise this pathway [43]. The finding that the expression of these transcription factors and their targets are upregulated in Sfrp1^-/-^ mice suggests a novel pathway in which loss of S*frp*1 enhances lipogenesis and may explain why heavier fat depots are observed in the Sfrp1^-/-^ animals. Fatty acids are critical for embryonic development and also for the development of cancer. Interestingly, palmitate, an end product of *de novo* lipid sythesis, is an important regulator of Wnt signaling [33]. Thus, *Sfrp*1 deficiency in some tissues may also regulate Wnt activity through indirect control of the *de novo* lipid sythesis transcriptional machinery. The regulation of *de novo* lipid sythesis by *Sfrp1* loss is a novel observation and future research will need to be carried out to determine whether palmitate production is higher in the tissues of Sfrp1^-/-^ mice.

We clearly demonstrate that loss of *Sfrp1* results in a marked effect on fasting glucose levels and glucose clearance (Figure 2A&B). Increased circulating glucose may be due to several factors including reduced insulin secretion, less β cells in pancreatic islets, insulin resistance, an impaired cellular ability to uptake glucose due to reduced glucose transporter levels, or overproduction of glucose in the liver by way of gluconeogenesis. Initially we hypothesized that Sfrp1^-/-^ animals produced a reduced amount of active insulin because our insulin tolerance tests showed that Sfrp1^-/-^ mice fed a HFD exhibited a very similar level of insulin resistance to control mice fed a HFD (Figure S2E). However, our insulin ELISAs revealed that the Sfrp1^-/-^ animals were in fact hyperinsulinemic on the HFD group as early as 2 weeks after starting the diet (Figure 2C). Interestingly, islets harvested from Sfrp1^-/-^ mice secreted significantly more insulin in response to glucose (Figure S2A) and these data help explain the observed elevated serum insulin levels in response to S*frp*1 loss. This is in agreement with the literature, as both Wnt3a and Wnt5a enhance glucose stimulated insulin release from islet cells and addition of recombinant human SFRP1 and SFRP4 has been shown to inhibit insulin secretion [44]. We also took a closer look at the islets and the insulin being released from Sfrp1^-/-^ mice and showed that insulin is cleaved properly and glucagon levels are not affected in response to Sfrp1 loss (Figure S2). However, although the number of pancreatic islets was increased in control mice fed a HFD as expected, this increase did not occur in response to S*frp*1 deficiency (Figure S2C) which could render Sfrp1^-/-^ mice more susceptible to β cell burn out and further metabolic dysregulation recapitulating a type-2 diabetic model. The reduction in glucose transporter expression (Figure 2D,E&F) also partially explains the observed hyperglycemia. Additional research must be undertaken to investigate the relationship between S*frp*1 loss and metabolic syndromes.

Obesity can also be associated with hepatosteatosis, which results from lipid accumulation in the liver and associated inflammation. Loss of *Sfrp1* results inhepatosteatosis (Figure 3A&B), which has been mechanistically demonstrated to be regulated by Il-6 as well as Ppar-α and Ppar-γ ligands leading to expression of *Fabp1*, *Chrebp* and other genes involved in *de novo* lipid sythesis [45,46]. However, S*frp*1 deficiency had very little effect on *de novo* lipid sythesis gene expression in the liver (Figure 3D) suggesting that the accumulated lipid is likely due to increased transport and accumulation of lipids from other organs. For instance, lipid accumulation can also occur in response to changes in the levels of insulin that signal through the insulin receptor substrate (IRS) which upregulate fatty acid transport proteins (FATP) that uptake and store circulating lipids made elsewhere in the body [47]. Thus, the systemic hyperinsulinemia together with the increased *Il-6* expression (Figure 3D) may also be driving the enhanced accumulation of lipid in the liver.

The increased macrophage infiltration and pro-inflammatory cytokine expression observed in Sfrp1^-/-^ mice was expected based on the link between obesity and inflammation. Periera et. al. have also demonstrated that human SFRP1 plays a role in cytokine regulation by showing that addition of recombinant human SFRP1 to LPS-stimulated macrophages results in a reduction in the levels of *Il-6, Il-8* and IL-1β[24]. However, the selective upregulation of Il-6 over *Tnf-*α and Il-1β in our Sfrp1^-/-^ mice (Figure 3D; Figure 4D; Figure 5B) differs from other models for Wnt antagonist loss. Specifically, Sfrp5^-/-^ mice exhibit an increase in the levels of all three inflammatory cytokines [21]. In addition, Mori et. al. showed that a compensatory increase in *Sfrp1* occurs in *Sfrp5* mutant mice and so we examined the levels of *Sfrp5, Sfrp4* and *Sfrp2* in gonadal adipose tissue and noted that expression of *Sfrp5* is increased in Sfrp1^-/-^ mice fed a HFD, while the control animals demonstrated a decrease in *Sfrp5* (data not shown). We suspect that some of the heterotypic effects that we observe in the gonadal cytokine expression levels may be due to the replacement and slightly differential activity of *Sfrp5*. 

Nicotinamide Phosphoribosyltransferase (Nampt *aka* Visfatin or Pbef) is an adipokine that has recently generated excitement since its expression has strong correlation with obesity and type-2 diabetes [47–50]. Interestingly, Nampt has also been shown to promote glucose-induced insulin secretion, protect against islet death, and regulate *Il-6* as well as *Il-17* expression in immune cells [51,52]. Moreover, *Nampt* is highly expressed in tumor cell lines and is required for several critical capabilities acquired during the process of tumorigenesis including *de novo* lipogenesis [53–56]. We consistently observed increased tissue expression of *Nampt* in Sfrp1^-/-^ mice (Figure 3D; Figure 4D; Figure 5B) which suggests that *Sfrp1* may be responsible for controlling the expression of *Nampt* and that this upregulation may explain the increased insulin secretion (Figure 2C) and *Il-6* expression (Figure 3D; Figure 4D; Figure 5B). Furthermore, an intermediate of Nampt, nicotinamide mononuceotide (Nmn) appears to suppress Il-1β which could contribute to our finding that Il-1β expression is repressed in the mammary gland of Sfrp1^-/-^ mice fed a HFD (Figure 5B) [57].

Previously, it has been demonstrated that suppression of SFRP1 expression is an early change in human premalignant breast lesions [58] and loss of *Sfrp1* in the mouse mammary gland results in hyperplastic lesions [36]. In this study we clearly demonstrate that the mammary glands of Sfrp1^-/-^ mice exhibit enhanced macrophage infiltration (Figure 5A;Figure S5). We also see elevated levels of *Tgf*-β2 in the mammary tissue in response to *Sfrp1* loss (Figure 5B). Tgf-β promotes the expression of pro-inflammatory mediators, including *Il-6* [59], which may partially explain the observed enhanced *Il-6* mRNA expression (Figure 5B). Additionally, in macrophages Tgf-β has been shown to suppressseveral cytokines, including *Il-1β* as well as *Tnf-α* depending on the context of stimulation [60–62] which supports our findings that Il-1β levels are reduced and *Tnf-α* is unaffected in Sfrp1^-/-^ mammary glands (Figure 5B). The expression profile of pro-inflammatory cytokines in response to *Sfrp1* loss and the HFD was not always consistant between the gonadal fat pad and the mammary gland which was expected considering that the immune responses differ in visceral and subcutaneos fat pads [63]. Finally, we detected a substantial increase in the expression of (Serpine1 *aka* PAI-1) (Figure 5B) which is involved in the regulation of cell adhesion, detachment and migration, thus playing an important role in cancer progression [64–66]. *Serpine1* is expressed in many types of cancer cells and allows the modulation of cancer growth, invasion and angiogenesis [67]. Moreover, a polymorphism in the human *SERPINE1* gene contributes to cancer risk [68,69]. Collectively, these data indicate that loss of *Sfrp1* may be a critical early event in obesity associated breast cancer initiation.

The typical western diet is rich in fat and carbohydrates and is resulting in rapid overall weight gain in the US population that has been likened to an epidemic. Several recent studies have noted an association between increased BMI and modulation of expression of several SFRP family members [70]. Furthermore, SNPs in the human *SFRP1* gene are associated with body composition suggesting that differences in *SFRP1* expression may play a role in regulating waist-to-hip ratios [71]. Thus, given the link between Wnt signaling, adipogenesis, diabetes and these epidemiological studies, it is important to ascertain whether SFRP family members are critical adipokines that can be used for the prevention of health issues related to weight gain and/or to identify higher risk individuals. This study focused on *Sfrp1* as it is expressed in and secreted from numerous tissuetypes. This research is important because it supports the notion that Sfrp1 is a critical secreted protein that is capable of regulating weight gain, glucose homeostasis and insulin production through distinct targets on various cell types. The alteration of expression of other secreted factors such as *Il-6*, *Nampt*, *Tgf-β* and *Serpine1* are likely to partially explain the pleiotropic effects observed and will be the focus of future studies.

## Supporting Information

Figure S1 **Tissue Weights** (A) Final organ weights from control and Sfrp1^-/-^ fed a ND for 12 weeks. (B) Final organ weights from control and Sfrp1^-/-^ fed a HFD for 12 weeks.
(TIF)

Figure S2 **Assessment of pancreatic function and morphology s in response to DIO and *Sfrp1* deficiency.** (A) Functional analysis of islets derived from control and Sfrp1^-/-^ mice was determined by glucose stimulated insulin secretion (GSIS). An Insulin ELISA was used to measure the concentration of insulin in the media of cultured islets stimulated with either low glucose (2.8 mM) or high glucose (16.7mM) for 2 hours. (B) Pancreas sections were subjected to immunohistochemical analysis and stained for Glucagon (brown chromogen) and representative images were captured at 200X are displayed for mice in each treatment group (scale bar 100 μm). (C) H&E stained pancreas slides were viewed at 40X and the total number of islets per tissue section was were counted. (D) Pancreas sections were subjected to immunohistochemical analysis and stained for C-peptide (brown chromogen) and representative images were captured at 200X are displayed for mice in each treatment group (scale bar 100 μm). (E) Insulin tolerance test (ITT) was performed at the end of the study. After a 4 h fast, mice were injected with 0.8U/kg BW insulin and blood glucose levels were monitored for 2 h. . (**p<0.01, significantly different from control mice fed a ND using Bonferroni’s *t* test after a two-way ANOVA.).
(TIF)

Figure S3 **Effect of *Sfrp1* deficiency on Wnt ligand expression in the liver.** Total RNA from the liver was used for real-time PCR analysis of *Wnt3a*, *Wnt5b*, and *Wnt10a* gene expression in mice from each treatment group (n=6). The results shown represent experiments performed in duplicate and normalized to the amplification of β-actin mRNA. (A) Data depict relative mRNA expression in ND fed control and Sfrp1^-/-^ mice. Bars represent mean ± SEM of the relative expression with respect to ND fed control mice. (B) Data depict relative mRNA expression in HFD fed control and Sfrp1^-/-^ mice. Bars represent mean ± SEM of the relative expression with respect to ND fed control mice. (^#^p<0.05, ^##^p<0.01, significantly different from respective ND fed mice using student’s *t* test.) .
(TIF)

Figure S4 **Effect of *Sfrp1* deficiency on Wnt ligand expression in the gonadal fat pad.** Total RNA from the gonadal fat pad was used for real-time PCR analysis of *Wnt3a*, *Wnt5b*, and *Wnt10a* gene expression in mice from each treatment group (n=6). The results shown represent experiments performed in duplicate and normalized to the amplification of β-actin mRNA. (A) Data depict relative mRNA expression in ND fed control and Sfrp1^-/-^ mice. Bars represent mean ± SEM of the relative expression with respect to ND fed control mice. (B) Data depict relative mRNA expression in HFD fed control and Sfrp1^-/-^ mice. Bars represent mean ± SEM of the relative expression with respect to ND fed control mice. (^#^p<0.05, ^##^p<0.01, significantly different from respective ND fed mice using student’s *t* test.) .
(TIF)

Figure S5 **Macrophage marker *Cd68* is elevated Sfrp1^-/-^ mice fed a HFD.** Total RNA from the mammary gland was used for real-time PCR analysis of *Cd68* in mice from each treatment group (n=6). The results shown represent experiments performed in duplicate and normalized to the amplification of β-actin mRNA. Bars represent mean ± SEM of the relative expression with respect to ND fed control mice. . (^#^p<0.05, significantly different from respective ND fed mice using student’s *t* test.) .
(TIF)

Figure S6 **Cononical Wnt signaling target gene, *Axin2*, is elevated in Sfrp1^-/-^ mice fed a HFD.** Total RNA from the mammary gland was used for real-time PCR analysis of *Axin2* in mice from each treatment group (n=6). The results shown represent experiments performed in duplicate and normalized to the amplification of β-actin mRNA. Bars represent mean ± SEM of the relative expression with respect to ND fed control mice. (**p<0.01, significantly different from control mice fed a ND using Bonferroni’s *t* test after a two-way ANOVA.).
(TIF)

File S1 **Supporting information materials and methods.** The experimental procedures utilized for generation of supplemental figures.
(PDF)

Table S1 **PCR primer sequences for real-time PCR analysis.**
(DOC)
