# Supplementary figures and images for: Mice Deficient in Sfrp1 Exhibit Increased Adiposity, Dysregulated Glucose Metabolism, and Enhanced Macrophage Infiltration
Source: PLoS One. 2013 Dec 5;8(12):e78320. doi: 10.1371/journal.pone.0078320 (PMC3855156; doi:10.1371/journal.pone.0078320)

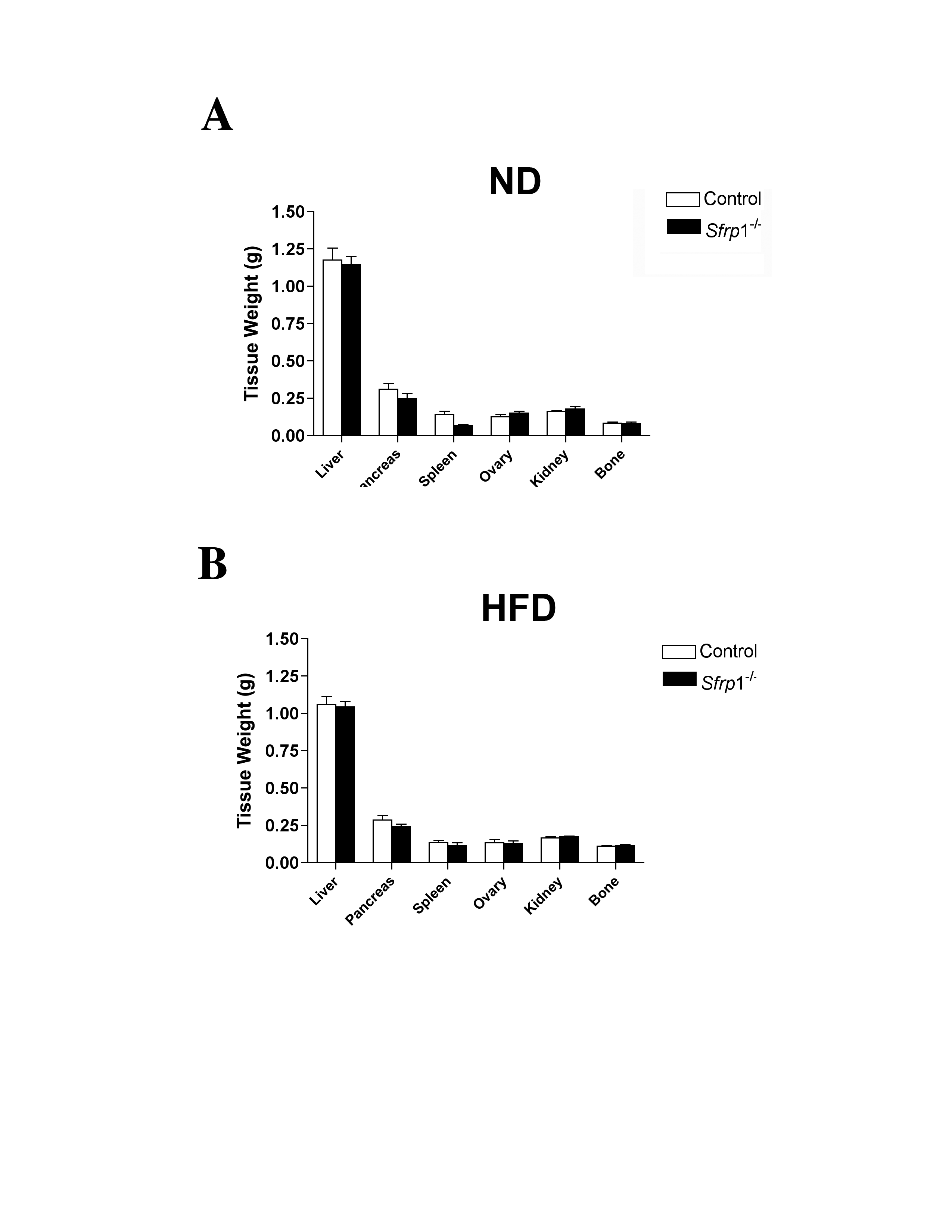

Supplement: Figure S1 — Tissue Weights (A) Final organ weights from control and Sfrp1-/- fed a ND for 12 weeks. (B) Final organ weights from control and Sfrp1-/- fed a HFD for 12 weeks. (TIF) [file pone.0078320.s001.tif]

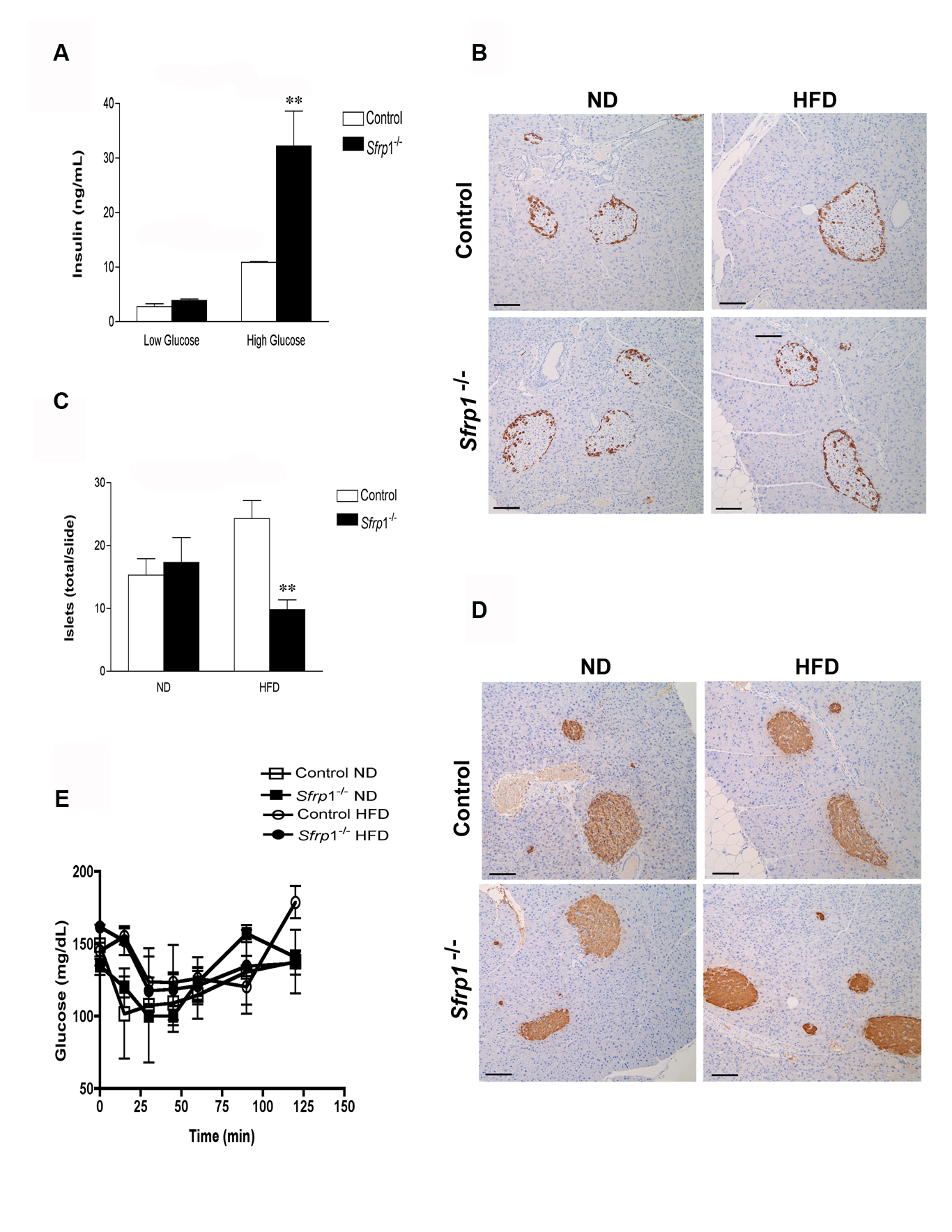

Supplement: Figure S2 — Assessment of pancreatic function and morphology s in response to DIO and Sfrp1 deficiency. (A) Functional analysis of islets derived from control and Sfrp1-/- mice was determined by glucose stimulated insulin secretion (GSIS). An Insulin ELISA was used to measure the concentration of insulin in the media of cultured islets stimulated with either low glucose (2.8 mM) or high glucose (16.7mM) for 2 hours. (B) Pancreas sections were subjected to immunohistochemical analysis and stained for Glucagon (brown chromogen) and representative images were captured at 200X are displayed for mice in each treatment group (scale bar 100 μm). (C) H&E stained pancreas slides were viewed at 40X and the total number of islets per tissue section was were counted. (D) Pancreas sections were subjected to immunohistochemical analysis and stained for C-peptide (brown chromogen) and representative images were captured at 200X are displayed for mice in each treatment group (scale bar 100 μm). (E) Insulin tolerance test (ITT) was performed at the end of the study. After a 4 h fast, mice were injected with 0.8U/kg BW insulin and blood glucose levels were monitored for 2 h. . (**p<0.01, significantly different from control mice fed a ND using Bonferroni’s t test after a two-way ANOVA.). (TIF) [file pone.0078320.s002.tif]

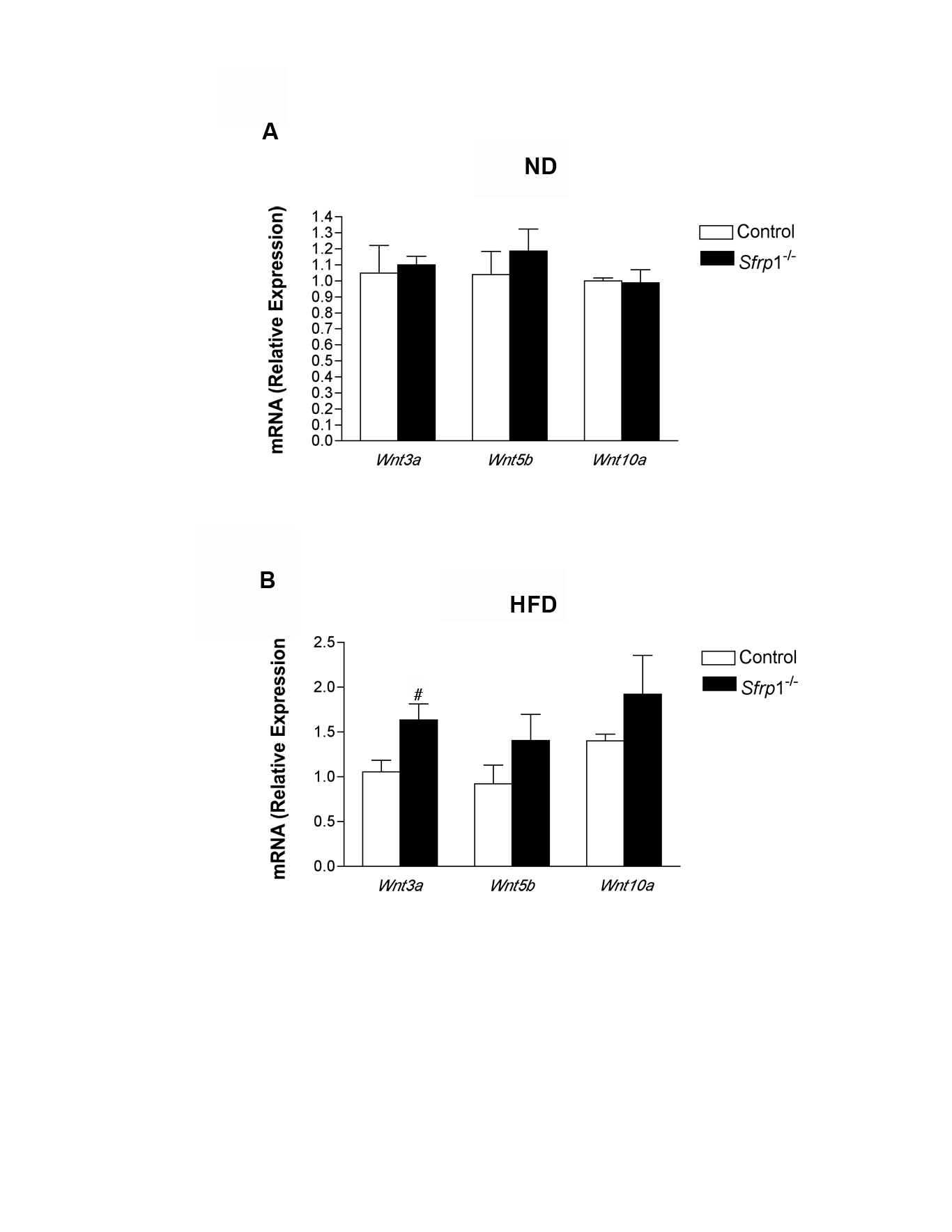

Supplement: Figure S3 — Effect of Sfrp1 deficiency on Wnt ligand expression in the liver. Total RNA from the liver was used for real-time PCR analysis of Wnt3a, Wnt5b, and Wnt10a gene expression in mice from each treatment group (n=6). The results shown represent experiments performed in duplicate and normalized to the amplification of β-actin mRNA. (A) Data depict relative mRNA expression in ND fed control and Sfrp1-/- mice. Bars represent mean ± SEM of the relative expression with respect to ND fed control mice. (B) Data depict relative mRNA expression in HFD fed control and Sfrp1-/- mice. Bars represent mean ± SEM of the relative expression with respect to ND fed control mice. (#p<0.05, ##p<0.01, significantly different from respective ND fed mice using student’s t test.) . (TIF) [file pone.0078320.s003.tif]

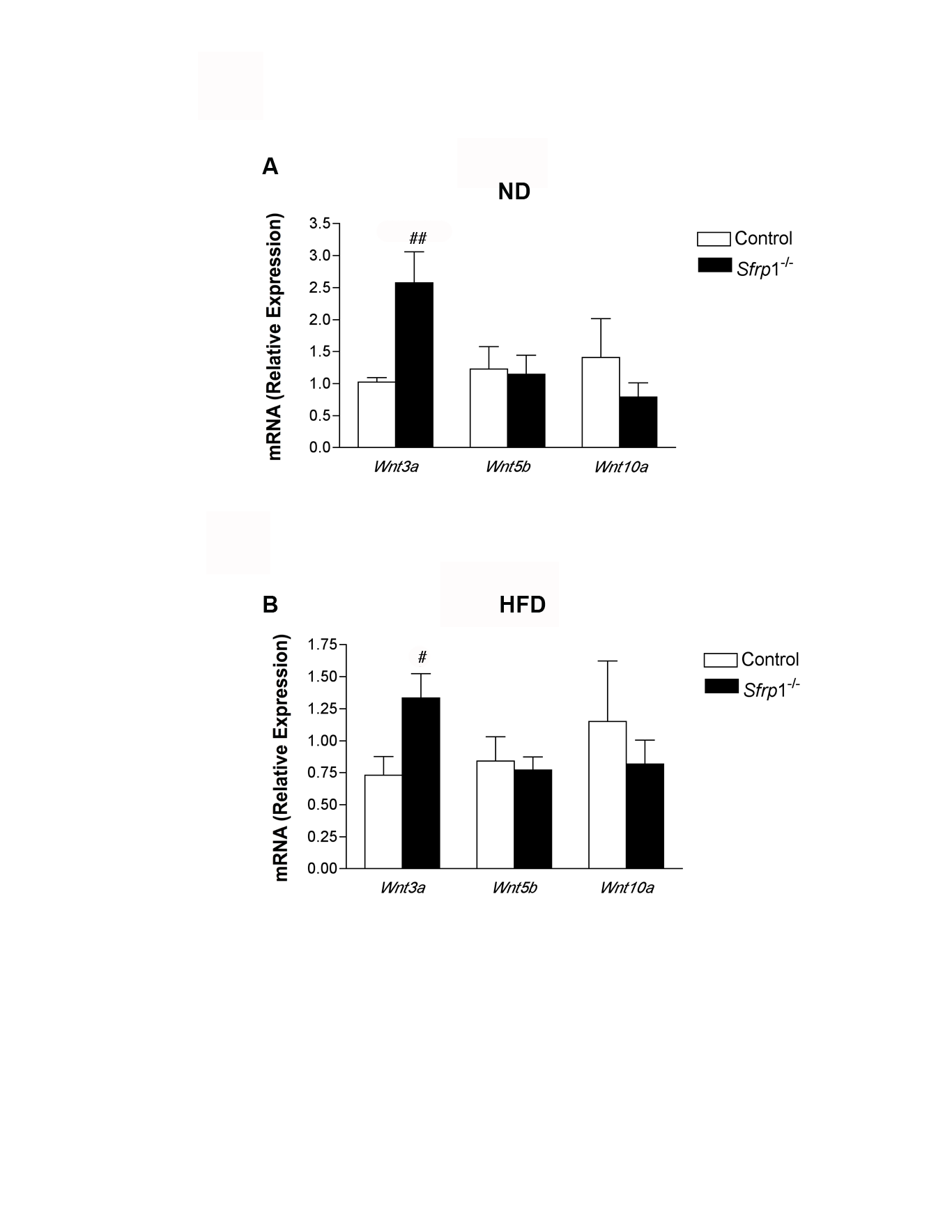

Supplement: Figure S4 — Effect of Sfrp1 deficiency on Wnt ligand expression in the gonadal fat pad. Total RNA from the gonadal fat pad was used for real-time PCR analysis of Wnt3a, Wnt5b, and Wnt10a gene expression in mice from each treatment group (n=6). The results shown represent experiments performed in duplicate and normalized to the amplification of β-actin mRNA. (A) Data depict relative mRNA expression in ND fed control and Sfrp1-/- mice. Bars represent mean ± SEM of the relative expression with respect to ND fed control mice. (B) Data depict relative mRNA expression in HFD fed control and Sfrp1-/- mice. Bars represent mean ± SEM of the relative expression with respect to ND fed control mice. (#p<0.05, ##p<0.01, significantly different from respective ND fed mice using student’s t test.) . (TIF) [file pone.0078320.s004.tif]

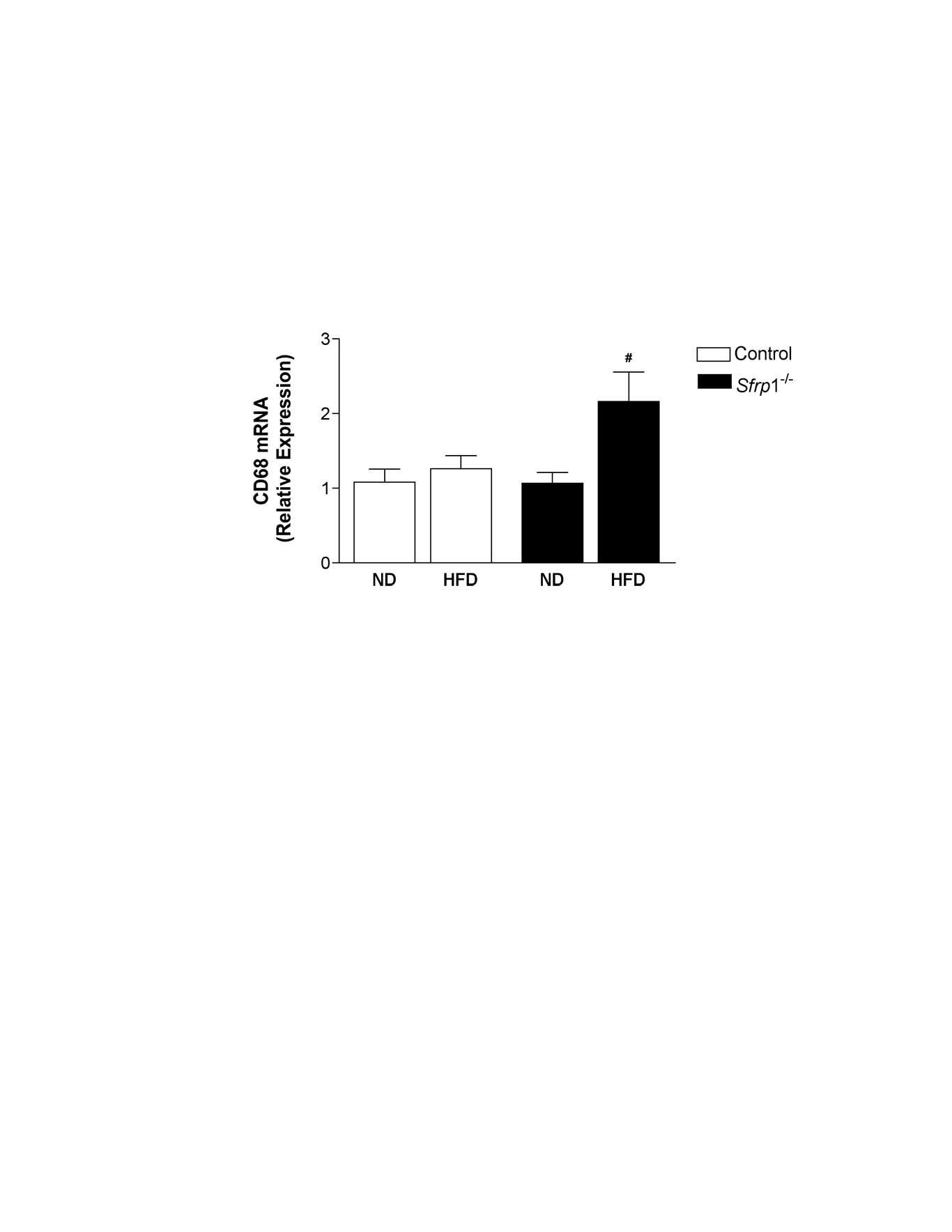

Supplement: Figure S5 — Macrophage marker Cd68 is elevated Sfrp1-/- mice fed a HFD. Total RNA from the mammary gland was used for real-time PCR analysis of Cd68 in mice from each treatment group (n=6). The results shown represent experiments performed in duplicate and normalized to the amplification of β-actin mRNA. Bars represent mean ± SEM of the relative expression with respect to ND fed control mice. . (#p<0.05, significantly different from respective ND fed mice using student’s t test.) . (TIF) [file pone.0078320.s005.tif]

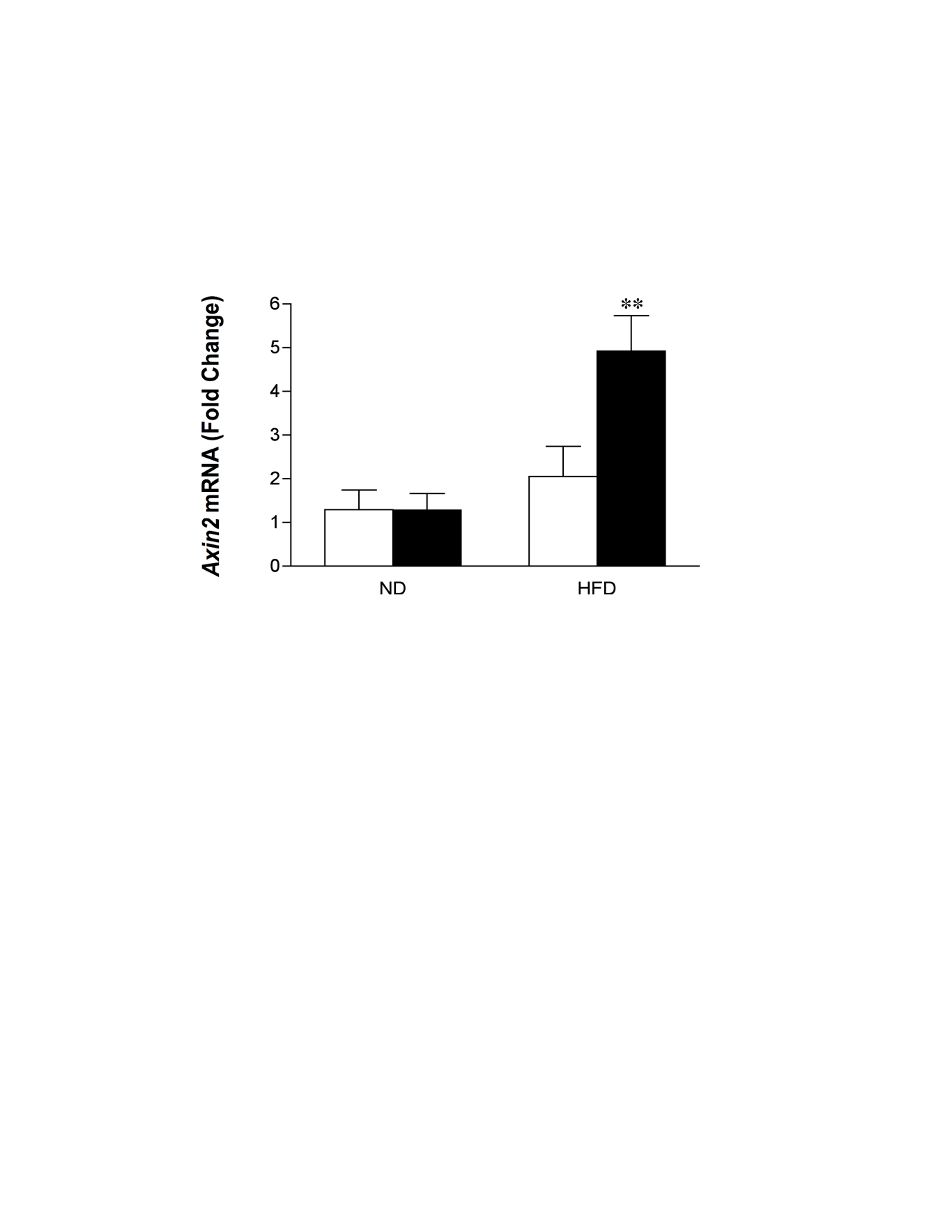

Supplement: Figure S6 — Cononical Wnt signaling target gene, Axin2, is elevated in Sfrp1-/- mice fed a HFD. Total RNA from the mammary gland was used for real-time PCR analysis of Axin2 in mice from each treatment group (n=6). The results shown represent experiments performed in duplicate and normalized to the amplification of β-actin mRNA. Bars represent mean ± SEM of the relative expression with respect to ND fed control mice. (**p<0.01, significantly different from control mice fed a ND using Bonferroni’s t test after a two-way ANOVA.). (TIF) [file pone.0078320.s006.tif]
