# Supplementary material for: Mice Deficient in Sfrp1 Exhibit Increased Adiposity, Dysregulated Glucose Metabolism, and Enhanced Macrophage Infiltration
Source: PLoS One. 2013 Dec 5;8(12):e78320. doi: 10.1371/journal.pone.0078320 (PMC3855156; doi:10.1371/journal.pone.0078320)
Supplement: File S1 — Supporting information materials and methods. The experimental procedures utilized for generation of supplemental figures. (PDF) [file pone.0078320.s007.pdf]

SUPPORTING INFORMATION MATERIALS AND METHODS

Islet Isolation

Age matched male129/C57Blk6 control mice (n=3) and 129C57Blk6 *Sfrp1*$^{-/-}$ mice (n=3) were sacrificed and Collagenase P enzyme solution (1.2-1.4 mg/mL; Roche Diagnostics Corporation, Indianapolis, IN) was injected to fully distend the donor pancreas. After digestion, islets were gradient purified and then handpicked. Cells were cultured with RPMI-1640 solution with 10% heat-inactivated fetal bovine serum and 1% penicillin-streptomycin (RPMI-1640, FBS and P-S all from Mediatech, Inc. Manassas, VA) in a 5% CO2 incubator overnight.

Glucose Stimulated Insulin Secretion Assay

Islets were equilibrated in low glucose solution (2.8mM) for one hour. Ten size matched islets (in triplicate samples) were placed in 12um Millicell Cell Culture PCF inserts (Millipore Corporation, Burlington, MA) in 24 well plates. Fresh, low glucose solution was added for 2 hours followed by moving the insert with the islets into high glucose solution (16.7mM) for 2 hours. Insulin secretion was determined by ELISA (Mercodia, Winston Salem, NC) according to the manufacturer's instruction.

Islet Quantification and IHC

Formalin fixed pancreas was paraffin-embedded and subjected to H&E staining (n=6/treatment group). Sections were viewed were captured with an Olympus BX41 light microscope and the total number of islets per tissue section were counted. IHC was

performed as described in the main body of the research article. Tissue was incubated with primary antibodies 1:100 [C-peptide (Cell Signaling, Danverse, MA) and Glucagon (Cell Signaling) for 45 minutes. Images were captured with an Olympus BX41 light microscope using SPOTSOFTWARE.

Insulin tolerance test

Mice were fasted for 4 hours, weighed, and their fasting blood glucose levels were measured from the tail vein using a glucometer (OneTouch Ultra; Lifescan, Milpitas, CA). Mice were injected with human regular insulin (Eli Lilly, Indianapolis, IN) at a concentration of 0.8 unit of insulin per kg of body weight in the interpretational cavity and blood glucose levels were assessed 15, 30, 60, 90, and 120 minutes after injection.

~~Cytokine Analysis~~

~~A multiplex elisa was utilized to obtain serum cytokine and chemokine levels. The assay was carried out with the Mouse UltraSensitive Pro-Inflammatory 7-plex Kit (Meso Scale Discovery, Rockville, MD) according to the manufacturer's instructions on a Meso Scale Discovery Sector Imager 2400A (Meso Scale Diagonsics, Rockville, MD).~~
