# Supplementary material for: Mice Deficient in Sfrp1 Exhibit Increased Adiposity, Dysregulated Glucose Metabolism, and Enhanced Macrophage Infiltration
Source: PLoS One. 2013 Dec 5;8(12):e78320. doi: 10.1371/journal.pone.0078320 (PMC3855156; doi:10.1371/journal.pone.0078320)
Supplement: Table S1 — PCR primer sequences for real-time PCR analysis. (DOC) [file pone.0078320.s008.doc]

| **Table S1: PCR primer sequences for real-time PCR analysis** | | |
| --- | --- | --- |
| *Wnt3a* | forward | 5’-CTTAGTGCTCTGCAGCCTGA-3’ |
|  | reverse | 5’-GGCCAGAAGCTCTGCTACTC-3’ |
|  |  |  |
| *Wnt5b* | forward | 5’-AACCATGTCTTTCTTGAGAGCAG-3’ |
|  | reverse | 5’- GGACCAACATCTTCCAAAGC- - 3’ |
|  |  |  |
| *Wnt10a* | forward | 5’- TCCTGTTCTTCCTACTGCTGCT -3’ |
|  | reverse | 5’- TCCTGTTCTTCCTACTGCTGCT-3’ |
|  |  |  |
| *Cd68* | forward | 5’-CTTCTGCTGTGGAAATGCAA -3’ |
|  | reverse | 5’- AGAGGGGCTGGTAGGTTGAT-3’ |
|  |  |  |
| *Axin2* | forward | 5’-CTGGCTCCAGAAGATCACAA -3’ |
|  | reverse | 5’-AGGTGACAACCAGCTCACTG-3’ |
